# Acorn Shell Extract-Functionalized Cotton Biomaterials: A Sustainable Platform with Biological Activities and Controlled Release for Skin Microbiota Dysregulation Conditions

**DOI:** 10.3390/pharmaceutics18101301

**Published:** 2026-10-14

**Authors:** Cláudia S. Oliveira, Andreia Fernandes, Adma Melo, Tiago B. Afonso, Ana F. Costa, Manuela Pintado, Carla Silva, Freni K. Tavaria

**Affiliations:** 1 CBQF—Centro de Biotecnologia e Química Fina—Laboratório Associado, Escola Superior de Biotecnologia, Universidade Católica Portuguesa, Rua Diogo Botelho 1327, 4169-005 Porto, Portugal; s-ansifernandes@ucp.pt (A.F.); admelo@ucp.pt (A.M.); tbafonso@ucp.pt (T.B.A.); mpintado@ucp.pt (M.P.); ftavaria@ucp.pt (F.K.T.); 2 RISE-Health—Faculdade de Medicina, Universidade do Porto, Alameda Prof. Hernâni Monteiro, 4200-319 Porto, Portugal; 3 LAQV—REQUIMTE, Departamento de Química e Bioquímica, Faculdade de Ciências, Universidade do Porto, Rua do Campo Alegre 687, 4169-007 Porto, Portugal; 4 Technological Centre for the Textile and Clothing Industries, CITEVE—Rua Fernando Mesquita 2785, 4760-034 Vila Nova de Famalicão, Portugal; afcosta@citeve.pt (A.F.C.); cjsilva@citeve.pt (C.S.)

**Keywords:** bioactive textiles, cotton functionalization, agri-food by-products, skin microbiota, phenolic compounds, skin compounds delivery

## Abstract

**Background/Objectives**: Skin dysbiosis is associated with several dermatological disorders and is often accompanied by oxidative stress and inflammation. Bioactive textiles offer a promising strategy for localized skin health management; however, many currently available materials rely on synthetic or metal-based agents. This study aimed to develop sustainable cotton-based bioactive textiles functionalized with acorn shell (AS) extract, an agri-food by-product rich in phenolic compounds, and evaluate their biological performance potential for skin-contact applications in the management of skin dysbiosis conditions. **Methods**: Cotton fabrics were pre-treated with chitosan, a commercial cationic agent, or potassium alum and functionalized with AS extract using exhaustion or padding techniques. The resulting materials were assessed for antimicrobial activity against skin-associated bacteria, antioxidant capacity, anti-inflammatory activity in LPS-stimulated macrophages, cytocompatibility with keratinocytes and macrophages, and phenolic compound release under skin-mimicking conditions. **Results**: AS-functionalized cotton exhibited strong antimicrobial activity, particularly against Gram-positive bacteria, together with significant antioxidant and anti-inflammatory effects. All functionalized textiles maintained keratinocyte viability above established cytocompatibility thresholds, supporting their suitability for direct skin contact. Exhaustion-treated samples showed higher phenolic incorporation and release, resulting in enhanced biological performance, whereas padding-treated fabrics exhibited lower phenolic loading, more sustained release profiles, and comparatively reduced bioactivity. **Conclusions**: AS extract can be successfully incorporated into cotton textiles using industrially relevant finishing methods, generating multifunctional materials with antimicrobial, antioxidant, and anti-inflammatory properties while maintaining cytocompatibility. These findings support the potential of AS-functionalized cotton as a sustainable, plant-based skin-contact textile platform for localized management of skin-dysbiosis-associated conditions, with great relevance for dermatological and healthcare applications.

## 1. Introduction

The human skin is a complex organ whose barrier integrity and immunological competence are tightly regulated by a dynamic and symbiotic relationship with its resident microbiota [1,2]. This skin–microbiota crosstalk plays a central role in maintaining homeostasis, protecting against pathogen colonization, and modulating local immune responses. Disruption of this equilibrium, commonly referred to as skin dysbiosis, is characterized by reduced microbial diversity, overgrowth of pathogenic species, oxidative stress, and chronic inflammation [3,4,5]. Such alterations are strongly associated with a broad spectrum of dermatological disorders, including atopic dermatitis (AD), psoriasis, acne, rosacea, hidradenitis suppurativa, impaired wound healing, and, in severe cases, skin cancer [6,7,8]. These conditions negatively impact quality of life and impose significant healthcare costs for millions of patients worldwide.

Current therapeutic strategies for dysbiosis-associated skin diseases rely largely on topical or systemic antibiotics, corticosteroids, and immunomodulators [9,10]. While often effective in the short term, these approaches are limited by high recurrence rates, adverse effects following prolonged use, and the accelerating emergence of antimicrobial resistance. These limitations highlight an urgent need for alternative strategies that are microbiota-friendly, locally acting, and compatible with long-term skin contact.

In this context, bioactive textiles have emerged as a particularly attractive approach for skin-directed interventions [1,11,12,13]. Due to their intimate, continuous, and prolonged contact with the skin, textile materials can act as reservoirs for bioactive compounds, enabling local delivery without systemic exposure. Antimicrobial textiles have shown promise in reducing pathogenic bacterial load on the skin surface, thereby contributing to microbiota rebalancing and symptom alleviation [1,13,14,15]. However, most available antimicrobial textiles are functionalized with synthetic chemicals or metal-based agents, such as silver or zinc oxide [14,16,17,18,19]. Despite their efficacy, these materials raise significant concerns regarding cytotoxicity, skin irritation, environmental persistence, and ecological impact, especially under repeated use and washing cycles. Consequently, the development of antimicrobial textiles based on natural, non-toxic, and sustainable compounds represents a critical step toward safer next-generation skin-interactive materials.

Agri-food by-products constitute an abundant, low-cost, and underexplored source of high-value bioactive molecules [20,21]. Although commonly treated as waste, residues derived from grains, legumes, fruits, and vegetables are often rich in phenolic compounds with well-documented antimicrobial, antioxidant, and anti-inflammatory activities [22,23,24]. As a result, such extracts are receiving increasing attention for applications in the pharmaceutical, cosmetic, and textile sectors, aligning with circular economy principles and sustainability-driven innovation [25,26,27]. In fact, their integration into textile substrates offers a dual advantage: valorisation of agri-food wastes and development of environmentally responsible functional materials.

Among these by-products, acorn shells (AS) from *Quercus* species stand out as a particularly promising resource. Typically discarded during animal feed processing, AS are exceptionally rich in phenolic compounds, including gallotannins, proanthocyanidins, gallic and ellagic acid derivatives, and flavonoids [20,28]. These molecules exhibit broad-spectrum biological activities relevant to skin health, such as antimicrobial effects against skin-associated pathogens, potent radical-scavenging capacity, and modulation of inflammatory signalling pathways [29,30,31,32,33]. Recent in vitro and in vivo studies have demonstrated that AS extracts exert antimicrobial, antioxidant, and anti-inflammatory effects in human-skin-related cellular and animal models, underscoring their potential for dermatological applications, particularly for AD lesions [20,33,34]. Nevertheless, despite this evidence, the biological performance of AS extracts following incorporation into textile materials remains unexplored.

From a materials perspective, bioextracts rich in phenolic compounds are attractive textile functionalization agents due to their intrinsic affinity for cellulose fibres [27]. Through hydrogen bonding, hydroxyl interactions, and metal-chelating capacity, these compounds can form stable complexes with cotton substrates, potentially enabling durable bioactivity. Moreover, tannins are known to exert multifunctional effects, combining antimicrobial action with antioxidant protection and anti-inflammatory properties [35,36]. Such a multifunctional profile is highly desirable for skin-contact textiles, where microbial control, oxidative stress mitigation, and immune compatibility must be addressed simultaneously.

Despite the growing interest in bioactive textiles incorporating natural extracts, important challenges remain regarding the development of sustainable materials capable of simultaneously providing antimicrobial, antioxidant, and anti-inflammatory activities while maintaining biocompatibility and controlled release of bioactive compounds. In particular, the influence of textile processing strategies on the incorporation, retention, release, and biological performance of plant-derived phenolic compounds remains insufficiently understood. Addressing these limitations is essential for the rational design of next-generation skin-interactive biomaterials intended for dermatological and healthcare applications.

In this context, the present study explores the valorization of AS, an underutilized agri-food by-product rich in phenolic compounds, as a sustainable source of bioactive components for cotton functionalization. Different surface pre-treatment strategies, such as cationization of the textile surface with chitosan (CH), a commercial cationic agent (CAT), and potassium alum (AL), combined with industrially relevant textile finishing methods were investigated to optimize the incorporation of AS extracts and evaluate their impact on the resulting material properties. The developed bioactive cotton textiles were comprehensively characterized in terms of antimicrobial, antioxidant, anti-inflammatory, and biocompatibility performance, while the loading and release of phenolic compounds were assessed under skin-mimicking physiological conditions. By establishing relationships between processing parameters, phenolic content, release behavior, and biological activity, this work provides new insights into the design of sustainable multifunctional biomaterials and advances the development of bioactive textiles for dermatological applications, including therapeutic garments and skin-health management strategies.

## 2. Materials and Methods

### 2.1. Preparation of Acorn Shell (AS) Extracts

Acorn shells (AS; *Quercus suber* L.) were kindly provided by Herdade do Freixo do Meio (Foros de Vale de Figueira, Montemor-o-Novo, Portugal). All procedures for the preparation of AS extracts were performed following a previously described methodology [20]. Briefly, upon collection, AS were stored in polyethylene bags, transported to the laboratory, and kept under controlled conditions until analysis. For the initial processing, the samples were homogenized, transferred to polyethylene flasks, and stored at −80 °C until further manipulation. Subsequently, AS were oven-dried at 55 °C until a moisture content of approximately 5% was achieved and then milled using a Thermomix TM5 kitchen robot (Vorwerk Elektrowerke GmbH & Co. KG, Wuppertal, Germany) to obtain a flour with a particle size of <1 mm. Finally, 100 g of each milled sample was stored in sampling bags in a dark, dry environment at room temperature (RT) until further use.

AS extracts were obtained using a hydroethanolic solvent system composed of 50% (*v*/*v*) absolute ethanol (ET; CH_3_CH_2_OH, Honeywell, Portugal, Porto) and 50% deionized water (H_2_O). Briefly, each sample was mixed with ET50/H_2_O50 at a solid-to-solvent ratio of 2:20 (*m*/*v*) and homogenized using an Ultraturrax T18 (Ika, Staufen, Germany) at 20,000 rpm for 1 min at 25 °C. The mixtures were then subjected to orbital shaking at 200 rpm for 60 min at 25 °C to promote solid–liquid extraction. After centrifugation at 10,000 rpm, the extracts were kept at 4 °C for 10 min and the supernatants were subsequently filtered through filter paper (4–7 μm, Prat Dumas, Couze St. Front, France) to remove residual solids. Ethanol was then evaporated at 37 °C using a Büchi R-210 rotary evaporator (BÜCHI Labortechnik AG, Flawil, Switzerland) and the resulting AS extracts were stored at 4 °C until the incorporation into textile materials.

### 2.2. Cotton Surface Modification and Functionalization with AS Extracts

The cotton knitted fabrics were first washed at 60 °C for 60 min in a domestic laundry machine using a non-ionic detergent (1 g/kg of textile) to eliminate possible contaminants. To enhance the adherence of the AS extract, three pre-treatment approaches were tested using agents that impart a positive charge (cationization) to the textile surface: two natural agents, chitosan (CH) (Qingdao Chibio Biotech Co., Ltd, Qingdao, China**)** and potassium alum (AL), and a synthetic commercial cationic agent, Denimcol Fix-GF (CAT). The pre-treatments with potassium AL and with the commercial cationic agent were performed using the exhaustion method in a Labomat BFA Mathis (Werner Mathis AG, Oberhasli, Switzerland). The commercial agent Denimcol Fix-GF (Tuebigen Chemical Company, Tübingen, Germany) was applied according to the supplier’s indications. The pre-treatment with potassium AL was performed by exhaustion using a solution with a concentration of 10 g/L alum for 30 min at 40 °C with 30 rpm. After pre-treatment, the fabrics were washed in the same conditions, with water at 30 °C for 20 min at 30 rpm. The pre-treatment with CH was applied by padding in a Foulard Mathis HVF 27291 (Werner Mathis AG, Oberhasli, Switzerland). This process consisted of impregnating the fabric for 5 min in an aqueous solution of 0.5% chitosan in 1% acetic acid. After that, the fabrics were passed through squeezing rolls, with a pressure of 4 bar and speed of 2 m/min. Finally, the samples were dried and cured for 3 min at 100 °C and 150 °C, respectively.

After pre-treatment, the samples were functionalized with AS extracts via exhaustion and padding techniques, which are the most common industrial textile processes. AS extract is a complex mixture of phenolic compounds and other constituents with different physicochemical properties [20]. Therefore, its deposition upon cotton is expected to occur predominantly through non-covalent interactions, including hydrogen bonding, polar interactions, adsorption phenomena, and physical retention within the fibre structure. Cotton is composed of cellulose, which contains a high density of hydroxyl groups capable of interacting with phenolic compounds through hydrogen bonding and hydroxyl-hydroxyl interactions.

Exhaustion is conventionally used for cotton fibres because their hydrophilic nature allows plant-derived compounds to diffuse into the fibre and interact through hydrogen bonding, improving uptake. It promotes the transfer of extract compounds from the aqueous treatment bath to the fibre, and the uptake depends on their solubility, polarity, molecular size and affinity for cellulose. The hydrophilic and more polar constituents are expected to be preferentially transported through the aqueous bath and interact with the hydroxyl groups of cellulose. Less polar or hydrophobic constituents may have lower affinity for the aqueous phase and for cellulose; nevertheless, their deposition can occur through adsorption at the fibre surface, association with other extract constituents deposited on the fibre, and physical retention within the fibre structure. Consequently, the result will be variable according to the affinities of the extract components for the fibre and treatment medium.

Padding ensures a more uniform distribution and enhances fixation on the fibre surface, compensating for the inherently weak binding of most plant extracts. In this process, the fabric is impregnated with the extract solution and subsequently squeezed, resulting in a controlled wet pick-up and a uniform distribution of the extract throughout the fabric. During subsequent drying and thermofixation, water removal increases the concentration of the extract constituents in the fibre surface and promotes their retention. Therefore, padding can facilitate the deposition of both polar and less-polar constituents present in the extract, although their final retention may differ according to their physicochemical properties.

The use of both processes was intended to investigate two established textile applications for functionalization with AS extract in cotton. This approach is particularly relevant for plant extracts, whose functional performance results from the combined contribution of several classes of compounds rather than from a single active component.

The functionalization by exhaustion was performed in the Labomat BFA Mathis (Werner Mathis AG, Oberhasli, Switzerland). A liquor ratio of 1:10 of the extract solution was used at 50 °C for 45 min at 30 rpm, with a heating gradient of 3 °C min^−1^. Additionally, an untreated cotton sample (the control) was functionalized in the previously mentioned conditions. In all the functionalizations, the remaining solution was discarded, and the samples were rinsed with water, squeezed, and air dried. The functionalization by padding was performed in the Foulard Mathis HVF 27291. For padding, the fabrics were submerged in the extract solution for 5 min and then passed through squeezing rolls, with a pressure of 2 bar and speed of 2 m/min. To finalize, the functionalized fabrics were thermofixed in a Mathis DHE 51991 (Werner Mathis AG, Oberhasli, Switzerland) for 5 min at 150 °C.

The different conditions of textile treatments were identified as follows: Blank cotton (untreated and non-functionalized cotton); cotton (control) + AS extract (untreated cotton functionalized with AS extract); cotton + CH pre-treatment + AS extract (chitosan pretreated cotton functionalized with AS extract; cotton + CAT pre-treatment + AS extract (cotton-pretreated with a commercial cationic agent and functionalized with AS extract); cotton + AL pre-treatment + AS extract (cotton pre-treated with potassium alum and functionalized with AS extract). Afterwards, to conduct the assays and prevent contamination, all textiles were cut into squares (±1 cm in length) and sterilized using UV light for 20 min on each side.

### 2.3. Evaluation of the AS-Functionalized Cotton Bioactivities

#### 2.3.1. Antimicrobial Activity

**Figure 1:**
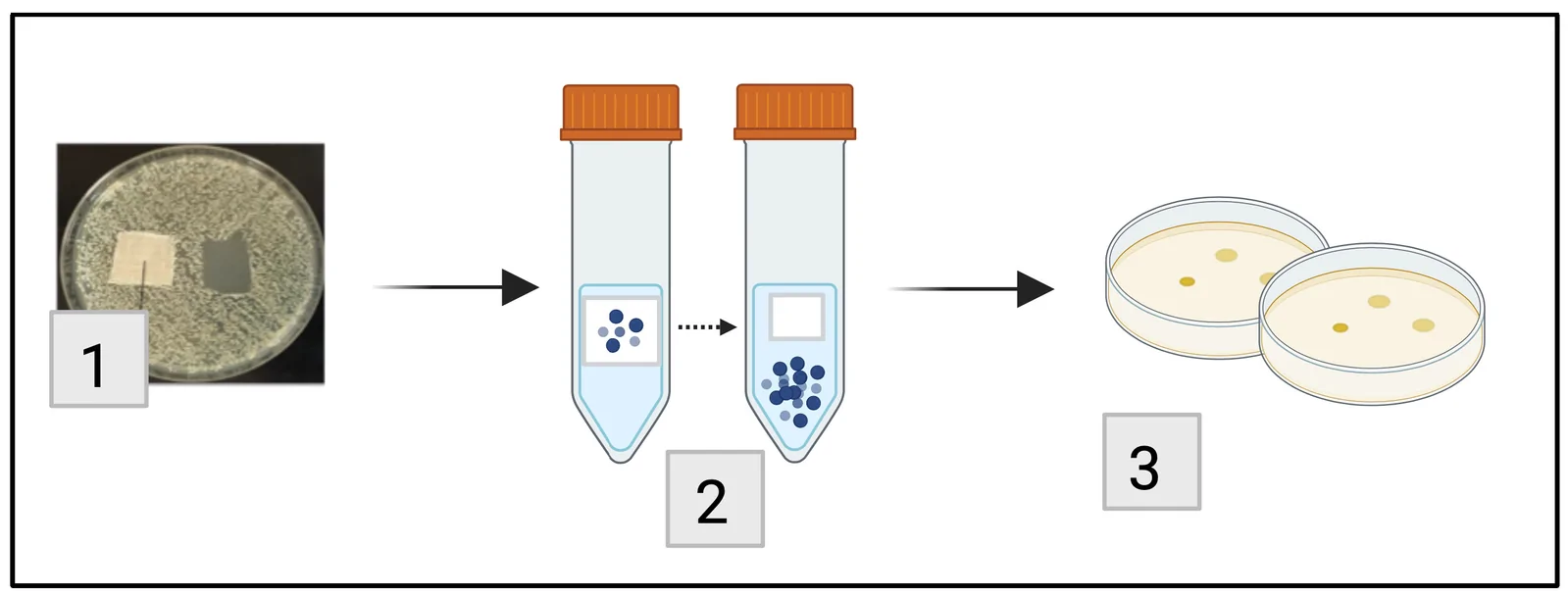
Experimental design for evaluation of the number of viable and attached bacteria on cotton samples. (1) After incubation at 37 °C, bacterial growth under the different cotton samples was assessed. (2) Each textile sample was removed, transferred to 2 mL of sterile saline solution, and vortexed to detach the bacteria from the fabric. (3) The resulting supernatant was serially diluted and plated on agar, and bacterial colonies were counted after incubation at 37 °C for 24 h.

The antimicrobial activity of AS-functionalized cotton was evaluated using a quantitative assay using a previously described methodology with some modifications [37]. Suspensions of *Staphylococcus aureus* (ATCC 6538), *Staphylococcus epidermidis* (DSM 20044), and *Escherichia coli* (ATCC 8739) were prepared in Tryptic Soy Broth (TSB, Biokar Diagnostics, Allonne, France) and incubated at 37 °C for 24 h. A sterile loop was used to extract a small sample from each bacterial suspension, which was then streaked onto Tryptic Soy Agar (TSA, Biokar Diagnostics). The streaked plates were incubated under optimal conditions until visible colonies formed. Subsequently, a single isolated colony from each plate was selected and inoculated into TSB, followed by incubation at 37 °C for approximately 24 h. Fresh inoculum for each bacterial strain was prepared from the previous inoculum for all assays. The bacterial concentration, i.e., colony-forming units (CFU)/mL of each inoculum was determined during the mid-log growth phase using densitometry and CFU counting to ensure accurate bacterial concentrations for the antimicrobial assays. The quantitative antibacterial assay was performed according to the guidelines of ISO 20645:2004 [38], with slight modifications (Figure 1). Briefly, 750 mL of each bacterial inoculum (concentration approximately 8 log CFU/mL) was added to a flask containing 100 mL of TSA heated to 45 °C. To cultivate a bacterial lawn, 5 mL of the bacterial suspension was evenly spread over 10 mL of pre-solidified TSA. After 10–15 min, three textile pieces per condition were aseptically placed on the agar surface using sterile tweezers. The agar plates were then incubated at 37 °C for 24 h. Afterwards, to evaluate bacterial adhesion to the different AS-functionalized cotton samples, we followed the methodology previously described [39] with some modifications. Briefly, after 24 h of incubation, each textile sample was retrieved from the agar plate and immersed in 2 mL of sterile Ringer solution (Biokar Diagnostics). The samples were then vortexed five times for approximately 5 s to detach the bacterial cells from the cotton samples. The resulting supernatant was serially diluted up to 10^−5^ and plated onto TSA. After an additional 24 h of incubation at 37 °C, the colonies were counted, and the bacterial count was expressed as log CFU/mL. This process allowed for the quantification of viable bacteria that had adhered to the textile materials.

#### 2.3.2. Antioxidant Activity

The antioxidant activity of AS-functionalized cotton samples was evaluated following a previously described protocol [39]. Briefly, textile samples (≈1 mg) were cut into small pieces and placed in 2 mL Eppendorf tubes. Subsequently, 2 mL of a 2,2-diphenyl-1-picrylhydrazyl (DPPH, Sigma-Aldrich—Merck, Darmstadt, Germany) methanolic solution with an absorbance of 0.600 ± 0.100 at 515 nm) methanolic solution was added to each tube, and the samples were incubated for 30 min at RT, protected from light. After incubation, absorbance was measured at 515 nm using a spectrophotometer. All analyses were performed in triplicate per condition of textile sample and Trolox (1000 μM, Sigma-Aldrich—Merck, Darmstadt, Germany) was used as the standard antioxidant for the preparation of the calibration curve. Radical scavenging activity was then expressed as percentage of inhibition.

### 2.4. Cell Culture Assays

#### 2.4.1. Cell Lines and Culture Conditions

For biocompatibility evaluation, we used an immortalized human keratinocyte cell line (HaCaT) obtained from Cell Lines Service (Oppenheim, Denmark) and macrophages differentiated from the human monocytic leukemia cell line (THP-1, TB 202, ATCC, Manassas, VA, USA). Before the assays, keratinocytes were cultured in Dulbecco’s Modified Eagle Medium (DMEM, high glucose), supplemented with 10% Fetal Bovine Serum (FBS, BioWest SAS, Nuaillé, France) and 1% Penicillin-Streptomycin-Fungizone solution (Pen-Strep, Lonza, Walkersville, MD, USA) and maintained in T75 flasks at 37 °C in a humidified atmosphere containing 5% CO_2_. THP-1 cells were cultured in RPMI-1640 Medium supplemented with 0.05 mM 2-mercaptoethanol (Gibco, Waltham, MA, USA), 10% FBS, and 1% Pen-Strep.

#### 2.4.2. Evaluation of Biocompatibility

The release of toxic substances from a textile material in contact with the skin is a primary trigger for irritation. Accordingly, cytotoxicity testing is essential to assess the irritation risk associated with such materials. In this study, to ensure a comprehensive evaluation of the potential cytotoxicity of AS-functionalized cotton, both indirect and semi-direct assessment methods were employed.

Indirect Exposure of HaCaT to AS-Functionalized Cotton: To assess cytotoxicity using an indirect method, we followed previously described methodologies with minor modifications [40,41]. Leachates were prepared by incubating the AS-functionalized cotton samples in a physiological solution that mimics the human sweat. This solution was prepared according to ISO 105-E04:2023 [42], being composed of L-histidine monohydrochloride (0.5 g/L), sodium chloride (5 g/L), and disodium hydrogen phosphate dihydrate (2.2 g/L), adjusted to pH 7.4. Prior to incubation, cotton samples were cut into round discs (approximately 1 cm diameter) and sterilized using UV light for 20 min on each side. Each disc was placed in an Eppendorf tube containing 1 mL of artificial sweat solution and incubated for 24 h at 37 °C in a humidified atmosphere with 5% CO_2_. One tube with the solution and without any cotton sample was used as negative control. Simultaneously, HaCaT were seeded in 96-well plate at a density of 1 × 10^5^ cells/mL and allowed to adhere under the same culture conditions. After incubation, the textile discs were removed, and the resulting leachates were collected. The HaCaT culture medium was the replaced with 100 µL of a mixture consisting of 67% supplemented DMEM and 33% leachate solution [42]. Plates were incubated for 8 and 24 h at 37 °C in a humidified atmosphere with 5% CO_2_.

Semi-direct Exposure of HaCaT to AS-Functionalized Cotton: To assess cytotoxicity using a semi-direct method, we used a similar methodology to that described by Oliveira et al. 2025 [39]. Briefly, this method was used to evaluate potential cytotoxicity in HaCaT and macrophages differentiated from THP-1 cells. HaCaT were seeded in a 24-well plate at a density of 1 × 10^5^ cells per well and allowed to adhere overnight at 37 °C in a humidified atmosphere with 5% CO_2_. Afterwards, the growth medium was replaced with fresh medium, and a transwell insert (BRAND) containing a membrane with 1.2 cm diameter and a pore size of 8 µm was placed into each well. A textile sample of 1 cm^2^ was placed inside each transwell insert above the cell’s monolayers, ensuring no direct contact with the cells. A positive control, consisting of cells in culture medium without exposure to any cotton fabric, was also included. To evaluate cytotoxicity, the plates were then incubated for 8 and 24 h at 37 °C in a humidified atmosphere with 5% CO_2_.

After 24 h of exposure with the textile discs, for both indirect and semi-direct methods, the metabolic activity of HaCaT was assessed using the 3-(4,5-dimethylthiazol-2-yl)-2,5-diphenyltetrazolium bromide (MTT) assay. Following incubation, the leachate solution, textile samples, inserts, and culture medium were removed. Fresh DMEM containing MTT solution (5 mg/mL) was added, and the cells were incubated for 3–4 h at 37 °C in a CO_2_ incubator. Subsequently, the medium was discarded, and formazan crystals were solubilized by adding 100 µL of DMSO to each well of the 96-well plates and 500 µL to the 24-well plates. The plates were gently agitated for 10 min at room temperature to ensure complete dissolution. Absorbance was then measured at 570 nm using a microplate reader (Synergy 4, Biotek Instruments, Inc., Winooski, VT, USA).

Cell viability was calculated as a percentage of the control. Each condition was tested in triplicate in three independent experiments for both the indirect and semi-direct methods.

Semi-direct Exposure of THP-1-Derived Macrophages to AS-Functionalized Cotton: THP-1 monocytes were cultured in RPMI 1640 medium, supplemented with 10% FBS, 1% Pen-Strep, and 0.05 mM 2-mercaptoethanol at 37 °C in 5% CO_2_. For differentiation, cells were seeded in 24-well culture plates (3 × 10^6^ cells/well) and treated with 50 ng/mL phorbol-12-myristate-13-acetate (PMA) for 48 h. After washing with PBS, macrophage-like cells were stimulated with 1 µg/mL lipopolysaccharide (LPS) for 24 h to induce a pro-inflammatory phenotype. Following LPS stimulation, the attached cells were washed to remove residual LPS and then exposed to AS-functionalized cotton using the same semi-direct contact system described for HaCaT cells. In addition to cells exposed to the cotton materials, the following controls were included: cells without LPS, cells with LPS, and cells treated with 1 mM of ibuprofen (positive anti-inflammatory control). Briefly, 1500 µL of complete medium was added per well, and cells were incubated with the textile samples for 24 h under standard culture conditions. Cell viability was assessed using the BD™ Cell Viability Kit (BD Biosciences, Milpitas, CA, USA) according to the manufacturer’s instructions. After exposure, supernatants were collected, adherent cells were washed, detached by trypsinization, centrifuged, and resuspended in 500 µL PBS. Cells were stained with propidium iodide (PI, 4 µL) and thiazole orange (TO, 2 µL) for 5 min at room temperature in the dark and analyzed using a BD Accuri™ C6 flow cytometer (BD Biosciences, Milpitas, CA, USA). Live (TO^+^/PI^−^), damaged (TO^+^/PI^+^), and non-viable (TO^−^/PI^+^) populations were identified based on FL1 and FL3 fluorescence signals.

#### 2.4.3. Evaluation of Anti-Inflammatory Activity

Anti-inflammatory activity was assessed using the same semi-direct exposure model described above for THP-1–derived macrophages. Briefly, following 24 h of semi-direct contact with AS-functionalized cotton samples, culture supernatants were collected and centrifuged at 200× *g* for 10 min at 4 °C to remove cellular debris. Cell monolayers were washed with cold PBS and lysed with 200 µL of cold distilled water per well. Total protein content was determined using a BCA protein assay kit (Thermo Scientific™ Pierce™, Waltham, MA, USA) according to the manufacturer’s instructions. The supernatants were used for the quantification of the release of pro-inflammatory cytokines, namely interleukin-6 (IL-6, Biolegend 430504, San Diego, CA, USA), interleukin-1β (IL-1β, Biolegend 437004, San Diego, CA, USA), and tumor necrosis factor-α (TNF-α, Biolegend 430204, San Diego, CA, USA) using enzyme-linked immunosorbent assay (ELISA) kits following the manufacturers’ instructions. Cytokine concentrations were normalized to total protein content and expressed as pg/µg protein.

### 2.5. Evaluation of Phenolic Compound Release

The release of phenolic compounds from the AS-functionalized cotton textiles was evaluated in a physiological solution mimicking human sweat, composed of L-histidine monohydrochloride (0.5 g/L), sodium chloride (5 g/L), and disodium hydrogen phosphate dihydrate (2.2 g/L), adjusted to pH 7.4. Prior to incubation, cotton samples were cut into approximately 1 cm^2^ pieces and sterilized using UV light for 20 min on each side. Each cotton sample was individually placed in 24-well plates containing 1.5 mL of artificial sweat solution and incubated for 24 h at 37 °C under static conditions. Wells without any cotton and wells with blank cotton were used as negative controls. After incubation, the textile samples were removed, and the resulting solutions (t_24_) were collected for further analysis. The amount of phenolic compounds released into the artificial sweat solution was quantified using the Folin–Ciocalteu method as described by Melo et al., 2024 [43]. Briefly, appropriate dilutions of each sample (30 µL) were mixed with Folin–Ciocalteu (Merck, Darmstadt, Germany) reagent (100 µL, 20% (*v*/*v*) and sodium carbonate (100 µL, 7.4% *w*/*v*). The mixture was then allowed to react in the dark at room temperature for 30 min. The absorbance was then measured at 765 nm (Synergy H1, Biotek, Winooski, VT, USA) in a 96-well microplate. Total phenolic content (TPC) was expressed as milligrams of gallic acid (GAE) per textile sample.

To determine the initial phenolic content of each functionalized cotton sample (t_0_), textile samples were subjected to solvent extraction using methanol: water (80:20, *v*/*v*). During each extraction cycle, samples were vortexed for 1 min and subsequently subjected to ultrasonic treatment for 15 min to enhance the release of phenolic compounds from the textile matrix. This procedure was repeated three times. After each step, the supernatants were collected, pooled, and analyzed using the same Folin–Ciocalteu procedure described above.

The antioxidant activity of both the textile extracts (t_0_) and the release media (t_24_) were further evaluated using the ABTS (2,2′-azinobis (3-ethylbenzothiazoline-6-sulphonic acid)) assay according to a previously described methodology [44]. Trolox was used as standard, and the results were expressed as micromoles of Trolox equivalents (µmol TE) per textile sample. The release of phenolic compounds was calculated as the percentage of total phenolics in the artificial sweat relative to the initial phenolic content of the textiles. For each condition, two independent textile samples were analyzed at each time point (t_0_ and t_24_).

### 2.6. Statistical Analysis

Data were analyzed using the GraphPad Prism 10 software and are presented as means ± standard deviation (SD). Quantitative data obtained from the antimicrobial, antioxidant, cytotoxicity, anti-inflammatory, and total phenolic content (TPC) assays were analyzed using one-way or two-way analysis of variance (ANOVA), as appropriate, followed by Tukey’s Honest Significant Difference (HSD) post hoc test for multiple comparisons. Statistical significance was defined as follows: *^, #^ = *p* < 0.05, **^, ##^ = *p* < 0.01, ***^, ###^ = *p* < 0.001, ****^, ####^ = *p* < 0.0001.

## 3. Results and Discussion

### 3.1. Antimicrobial Activity of AS-Functionalized Cotton

To corroborate the antimicrobial potential of AS extracts against clinically relevant skin-associated bacteria, we evaluated the antibacterial activity of AS-functionalized cotton samples against two major skin pathogens (*Staphylococcus aureus* and *Escherichia coli*), as well as a commensal member of the human skin microbiota, *Staphylococcus epidermidis*. In this context, we aimed to determine how effectively AS extracts inhibit bacterial growth when incorporated into cotton using either the exhaustion or padding method across different surface pre-treatments. This approach allowed us to compare the antimicrobial performance of the developed AS-functionalized cotton materials and to understand how processing conditions influence their biological efficacy.

**Figure 2:**
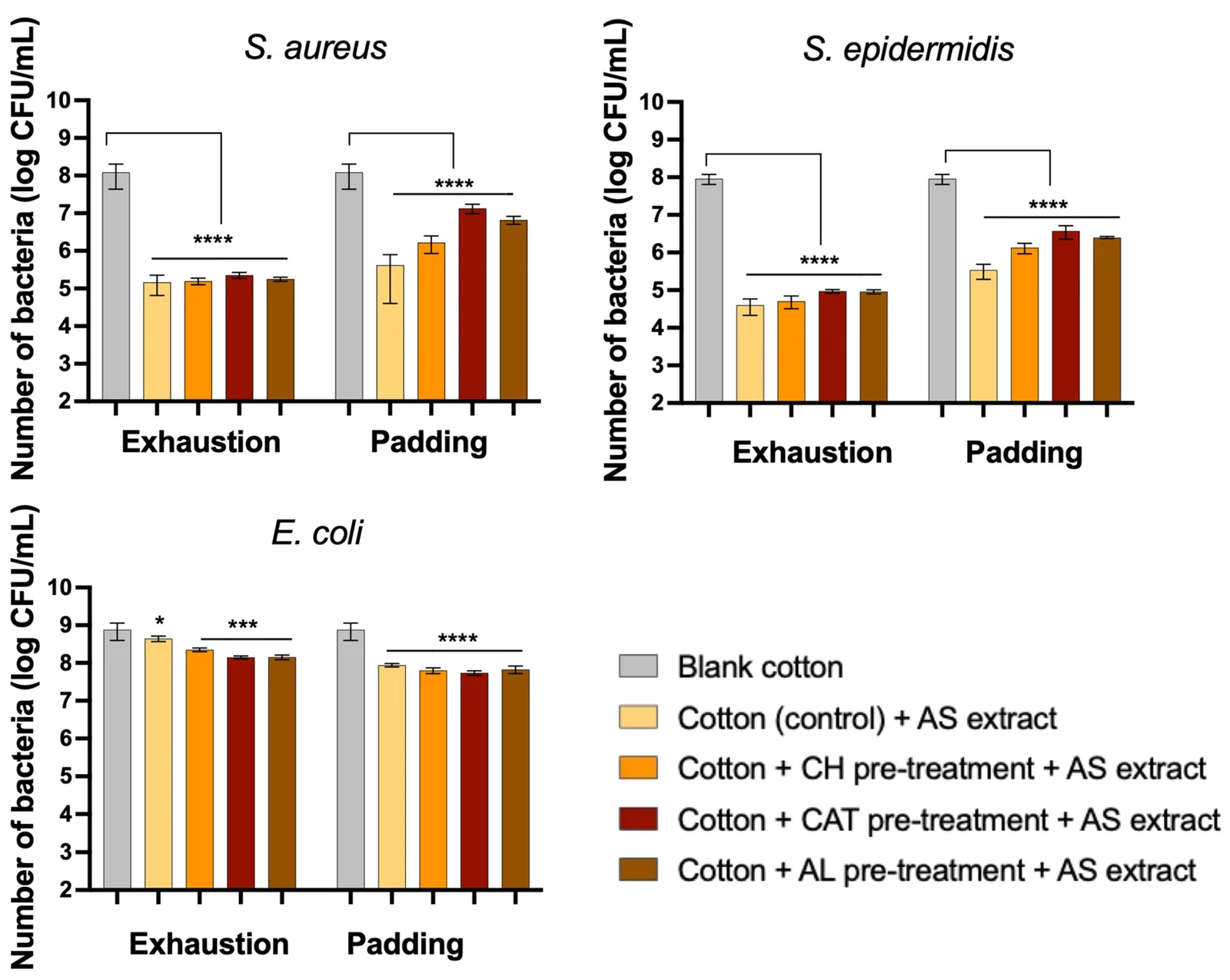
Quantification of viable *S. aureus*, *S. epidermidis*, and *E. coli* adsorbed onto the textile specimens. Data are presented as the mean of three textile samples per condition. Statistically significant differences are indicated as follows: * = *p* < 0.05, *** = *p* < 0.001, and **** = *p* < 0.0001, relative to blank cotton. Blank cotton (untreated and non-functionalized cotton); cotton (control) + AS extract (untreated cotton functionalized with AS extract); cotton + CH pre-treatment + AS extract (chitosan pretreated cotton functionalized with AS extract); cotton + CAT pre-treatment + AS extract (cotton pre-treated with a commercial cationic agent and functionalized with AS extract); cotton + AL pre-treatment + AS extract (cotton pre-treated with alum and functionalized with AS extract).

To this end, after 24 h of direct contact between the bacteria and the textiles, we quantified the number of viable bacteria attached to each of the AS-functionalized cotton materials (Figure 1). For *S. aureus*, the blank cotton displayed very high bacterial loads (approximately 10^8^–10^9^ CFU/mL) as demonstrated in Figure 2. In contrast, all AS-treated textiles prepared by the exhaustion method, independently of the pre-treatment applied (control, CH, CAT, or AL), showed a strong antibacterial effect, reducing bacterial counts to ~10^5^ CFU/mL (*p* ≤ 0.0001). The padding method also led to a reduction in viable bacteria in comparison to blank cotton, although to a lesser extent, with counts remaining around ~10^6–^10^7^ CFU/mL (*p* ≤ 0.0001) across all pre-treatment conditions.

A similar behavior was observed for *S. epidermidis*, where blank cotton maintained high bacterial loads close to 10^8^ CFU/mL. In contrast, the AS-functionalized cotton samples produced by the exhaustion method induced a pronounced reduction in bacterial growth, decreasing counts to ~10^4^–10^5^ CFU/mL (*p* ≤ 0.0001) (Figure 2). AS-functionalized cotton prepared by padding exhibited moderate antibacterial activity, reducing bacterial counts to approximately 10^6^ CFU/mL (*p* ≤ 0.0001). In the case of *E. coli*, blank cotton reached bacterial loads of about 10^9^ CFU/mL, while AS-treated textiles produced by the exhaustion method displayed a modest but significant antibacterial effect, particularly for CH, CAT, and AL pre-treated samples, reducing the viable bacteria to ~10^8^ CFU/mL (*p* ≤ 0.001) (Figure 2). When the padding method was used, reductions were slightly more pronounced, and all AS-functionalized samples showed significantly lower bacterial levels compared with blank cotton (*p* ≤ 0.001).

The strong antibacterial activity observed for AS-functionalized cotton samples is consistent with the high phenolic and tannin content of AS extracts [20,28,43]. Previous chemical analyses of *Quercus* AS extracts identified gallotannins, condensed tannins (proanthocyanidins), gallic/ellagic acid derivatives and other phenolic acids and flavonoids as dominant constituents [20,28]. These compounds are widely associated with antimicrobial and antioxidant properties.

It is known that phenolic acid-rich extracts exert antimicrobial effects through multiple complementary actions: binding to and disrupting microbial cell envelopes, complexing with surface and intracellular proteins (including enzymes), depriving microbes of essential metal ions, and interfering with biofilm formation and adhesion [28,45]. These multi-target effects commonly translate into greater efficacy against Gram-positive species, which lack the additional outer membrane barrier present in Gram-negative bacteria.

Such differences in susceptibility provide a plausible explanation for our observations: *S. aureus* and *S. epidermidis* (Gram-positive) were reduced by several logs when in contact with AS-functionalized cotton, whereas reductions in *E. coli* (Gram-negative) were more modest.

The strong antimicrobial activity observed in our study when the AS extract is incorporated by exhaustion method may reflect differences in extract uptake, distribution, and retention within the fibre structure. Exhaustion (a bath-based impregnation under agitation) typically promotes deeper adsorption and greater loading of hydrophilic polyphenols into cellulose and fibrillar micro-pockets, whereas padding tends to deposit more surface-localized material that can be less distributed and more prone to rapid leaching. These process-dependent differences in loading and release kinetics would be expected to amplify antimicrobial effects for exhaustion-treated samples and are in line with a study comparing textile functionalization strategies [46]. In addition, surface pre-treatment (e.g., through cationization, crosslinkers or plasma/chemical pre-treatments) can increase affinity for anionic polyphenols and tannins, improving adsorption and durability [47]. This correlates with the enhanced activity observed for certain pre-treated + exhaustion conditions in our data.

Next, to further assess the cotton surface after functionalizations, scanning electron microscopy (SEM) was performed for all samples in study. Representative SEM images revealed morphological differences between blank cotton and AS-functionalized samples, confirming the successful deposition of the AS extract onto the fibre surface, regardless of the functionalization method employed (Figure S1, Supplementary Materials). However, some variations in surface morphology were observed among different pre-treatments, suggesting differences in AS extract incorporation and distribution.

In this sense, to better understand the correlation between the incorporation of AS extract in cotton surface, different methods of functionalization, pre-treatments, and biological performance, future studies should explore, for example, the release of key bioactive components from AS-cotton functionalized samples, namely phenolic compounds and tannins, under physiologically relevant conditions of the skin.

The pronounced antibacterial activity of AS-functionalized cotton against Gram-positive bacteria, but lesser extent against Gram-negative species, is consistent with previous studies incorporating plant-derived extracts into textile materials [37,39,48]. This selective property supports the rationale for using such bioactive textiles to specifically target Gram-positive bacteria. This supports their potential application in therapeutic garments or dressings designed for dermatological conditions where *S*. *aureus* overgrowth plays a key pathogenic role, such as atopic dermatitis (AD).

In this line, clinical evidence shows that antibacterial textiles can reduce *S. aureus* skin colonization and, in some studies, lead to clinical improvement: silver-coated garments produced rapid and significant reductions in *S. aureus* density and were associated with symptomatic improvement in patients with AD [14,15,49]. However, advancing AS-functionalized textiles into clinical trials requires demonstrating several key attributes beyond their in vitro antimicrobial potency. These include: (i) the ability to significantly reduce *S. aureus* colonization using both three-dimensional (3D) human skin models and in vivo models; (ii) a favorable safety and tolerability profile, with no skin irritation, sensitization, or adverse reactions; and (iii) preliminary evidence that skin microbiota modulation can drive meaningful improvements in disease severity, flare frequency, or patient-reported outcomes.

Additionally, to broaden the biomedical application of these materials, it will be necessary to enhance their antibacterial activity against Gram-negative bacteria, particularly *E. coli*. The lipopolysaccharide-rich barrier of Gram-negative bacteria restricts the penetration of bulky or hydrophilic bioactive molecules, limiting their access to intracellular targets and frequently necessitating higher concentrations or synergistic strategies to achieve bactericidal effects [50].

To address this challenge, several studies have shown that the antibacterial activity against Gram-negative bacteria of plant-derived extracts can be enhanced when combined with complementary antimicrobial agents, including metal-based nanoparticles (e.g., Ag, Cu, ZnO), CH coatings, or essential oils such as tea tree, thyme, and oregano, which act as membrane disruptors [51,52]. Consistent with this, the slight inhibition of *E. coli* growth observed following pre-treatment with CH, CAT, and AL in AS-functionalized cotton produced via the exhaustion method suggests that the intrinsic activity of the incorporated compounds remains limited by insufficient penetration or interaction with the bacterial envelope, highlighting the need for optimized formulations that enhance delivery efficiency and/or exploit synergistic mechanisms to achieve more pronounced antibacterial effects against Gram-negative bacteria.

In conclusion, AS-functionalized cotton—particularly when applied by exhaustion and with appropriate surface pre-treatments—shows robust activity against Gram-positive skin pathogens and moderate activity against Gram-negative *E. coli*. These findings are consistent with the known chemistry and mechanisms of phenolic compounds from AS extracts and, when considered alongside the limited penetration through Gram-negative outer membranes discussed above, underscore the need for further optimization and mechanistic investigation—particularly through strategies that enhance compound delivery and exploit synergistic interactions—to advance the development of safe and effective bioactive textiles for skin applications.

### 3.2. Antioxidant Activity of AS-Functionalized Cotton

Skin is continually exposed to environmental stressors, namely UV radiation, pollution, chemicals, and pathogens, that can generate reactive oxygen species (ROS). Excessive ROS contribute to inflammation, pigmentation and aging, impaired barrier function, delayed tissue repair, and are also associated with several dermatological disorders, including those correlated with skin microbiota dysregulation [53,54]. In fact, in a skin microenvironment of dysbiosis, alterations in microbiota composition and host-microbiota interactions my contribute to an imbalance between ROS production and antioxidant defense, resulting in increased oxidative stress that can further compromise skin homeostasis and barrier integrity. Therefore, textile materials capable of neutralizing ROS at the skin-textile surface represent a promising strategy to mitigate oxidative damage while supporting skin homeostasis and tissue recovery [55].

In this context, given the strong antioxidant activity of AS extracts reported in in vitro studies, we sought to investigate whether this biological property is retained after their incorporation into cotton materials. Consequently, we expected that the AS-functionalized cotton may act as a reservoir of bioactive molecules capable of scavenging ROS at the skin–textile interface.

**Figure 3:**
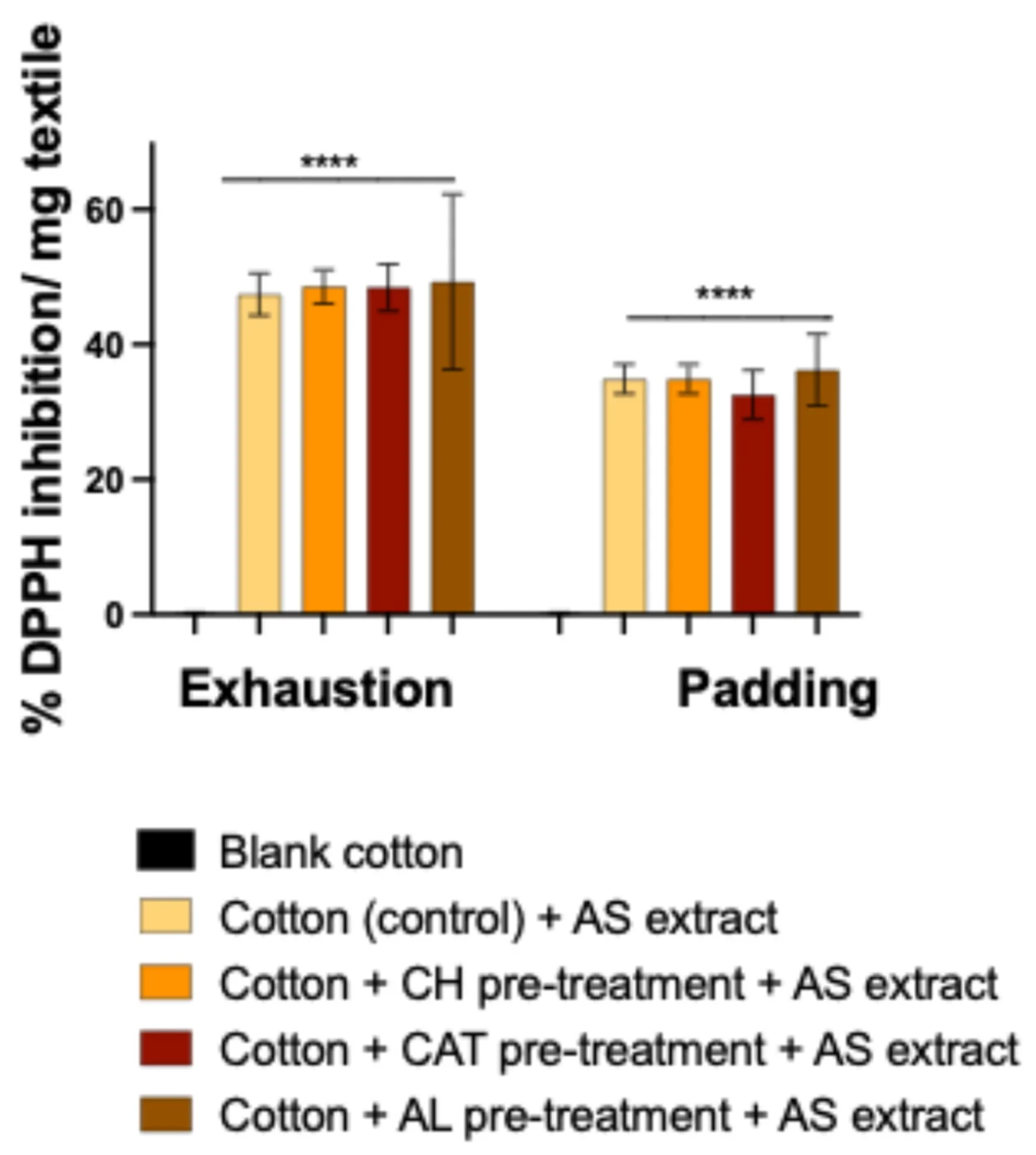
Antioxidant activity of AS-functionalized cotton samples demonstrated by exhaustion and padding finishing methods. Percentage of DPPH inhibition per milligram of textile for blank cotton (untreated cotton samples), AS-treated cotton without any surface pre-treatment (cotton control + AS extract) and AS-functionalized with different surface pre-treatments (CH, CAT, AL). Samples finished by exhaustion exhibited higher antioxidant activity compared with those finished by padding. Pre-treatments did not significantly affect the antioxidant performance within each finishing method. Data are presented as mean ± SD of three independent experiments performed in triplicate. **** *p* < 0.0001 versus cotton blank.

The antioxidant activity of the AS-functionalized cotton samples was evaluated by quantifying the percentage of DPPH inhibition per milligram of textile. In general, the exhaustion technique resulted in higher antioxidant performance compared with the padding (Figure 3). More specifically, cotton samples finished by exhaustion exhibited inhibition values between approximately 45–50%, demonstrating an efficient incorporation of the AS extract. The inclusion of different pre-treatments (CH, CAT, or AL) did not substantially alter the antioxidant activity, as all samples, including cotton control (with AS extract but without any surface pre-treatment), showed similar inhibition levels when compared to blank cotton (untreated cotton) (*p* ≤ 0.0001). Although the AL pre-treatment displayed slightly greater variability, its mean value remained comparable to the other conditions.

In contrast, when using the padding technique for the incorporation of AS extracts, we observed lower antioxidant activity, with DPPH inhibition ranging from approximately 33–38%. Similar to the exhaustion technique, the application of CH, CAT, or AL pre-treatments did not markedly influence the antioxidant performance of the AS-treated textiles, as the mean values remained similar across the different conditions. Despite the lower efficiency of this finishing technique, all padding-treated samples still demonstrated significantly higher antioxidant activity compared to blank cotton (*p* ≤ 0.0001).

Together, these findings indicate that the exhaustion method is more effective than padding for enhancing the antioxidant properties of cotton using the AS extract. Moreover, the results show that the selected pre-treatments do not significantly impact the antioxidant activity achieved with either finishing method.

As mentioned in the previous section, AS extracts are well recognized for their strong antioxidant activity due to their rich content in phenolic compounds, including gallic acid, ellagic acid, catechins, tannins, and other flavonoids. Several studies have demonstrated the potent radical-scavenging activity of *Quercus* spp. shell extracts using DPPH, ABTS, and FRAP assays, often reporting inhibition values comparable to or stronger than synthetic antioxidants [43,56,57]. These chemical constituents are also compatible with textile substrates due to their affinity for cellulose through hydrogen bonding, hydroxyl interactions, and tannin–fibre complexation, making AS extract an attractive bio-based finishing agent, particularly for cotton materials.

In the present study, AS-functionalized cotton exhibited notable antioxidant activity, confirming that the extract retained its radical-scavenging properties following textile incorporation. The exhaustion method consistently led to higher DPPH inhibition (≈45–50%) compared with the padding technique (≈33–38%). These results can be directly correlated with the possible distinct incorporation mechanism of each method: while exhaustion promotes deeper penetration and a more uniform distribution of phenolic compounds within cellulose fibres, padding typically leads to more superficial deposition and lower uptake efficiency. Consequently, the enhanced antioxidant performance of exhaustion-treated samples probably reflects a higher phenolic loading, accessibility and retention of bioactive compounds within the textile matrix.

Interestingly, the surface pre-treatments applied prior to AS incorporation (cationization via CH, CAT, or AL) did not significantly modify the antioxidant properties across either finishing method. This observation suggests that the antioxidant activity is primarily governed by the amount of extract absorbed rather than by chemical modifications of the cotton backbone. Similar results have been reported for other plant-derived phenolic finishes, where the intrinsic antioxidant potential of the extract—rather than fibre activation—was the dominant determinant of textile performance [52,55]. Although the AL pre-treatment introduced some variability, the overall mean inhibition remained comparable to other conditions, indicating that none of the pre-treatments substantially impaired or enhanced the polyphenol uptake at the levels achieved here.

Importantly, all AS-functionalized textiles, regardless of finishing method or pre-treatment, demonstrated significantly higher antioxidant activity than blank cotton, which exhibited minimal DPPH inhibition. This finding confirms the successful incorporation and functional retention of bioactive compounds from AS extract to the textile substrate.

Given that several phenolic compounds are well known for their combined radical-scavenging and antimicrobial properties, the enhanced antioxidant performance observed can be associated with their presence and activity within the fabric, supporting the potential of AS-functionalized cotton to deliver multifunctional benefits when applied to human skin.

### 3.3. Biocompatibility of AS-Functionalized Cotton

Bioactive textiles designed for direct skin applications must be rigorously assessed using physiologically relevant cell models to ensure both safety and functional performance. Keratinocytes form the outermost layer of the human skin and are the first cells in direct contact with external materials, including textiles, thus acting as physical and immune barrier [1]. Therefore, these cells are indispensable for evaluating potential skin cytotoxicity. Likewise, macrophages represent key immune regulators within the skin, responsible for detecting external stimuli, driving inflammatory responses, and coordinating tissue repair [58]. As a result, the inclusion of these distinct cell models is crucial to elucidate the impact of bioactive textiles on the viability of human skin cells. Moreover, evaluating different methods and time points for assessing cell viability may provide deep insights into both short- and prolonged-contact effects of AS-functionalized cotton materials.

**Figure 4:**
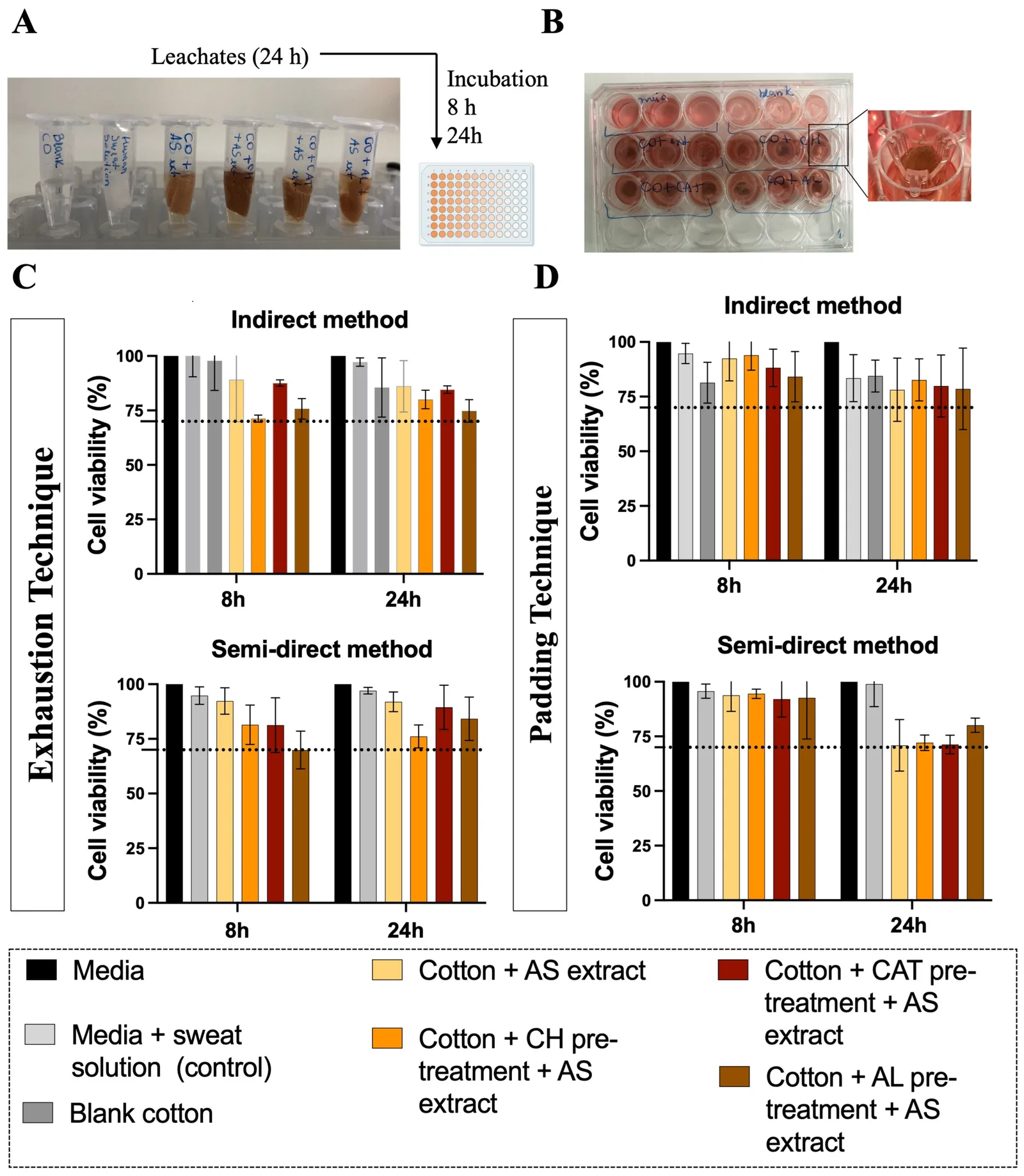
Cell viability of keratinocytes in the absence and presence of cotton samples (non-functionalized and functionalized with AS extracts) using indirect and semi-direct methods. (**A**) Schematic representation of the indirect method, where leachates were prepared from all cotton samples during 24 h of incubation. The cell viability of keratinocytes exposed to these leachates was assessed after 8 and 24 h of incubation in contact with artificial human sweat solution. (**B**) Schematic representation of the semi-direct method, where cotton samples were placed in inserts with an 8 μm pore membrane in a 24-well plate. The cell viability of keratinocytes exposed to the cotton samples was assessed after 8 and 24 h of incubation. (**C**,**D**) The percentage of the cell viability are represented for all the AS-functionalized cotton samples obtained by exhaustion and padding technique. Media (keratinocytes maintained only with cell culture media, without any cotton sample); Blank cotton (untreated and non-functionalized cotton); cotton (control) + AS extract (untreated cotton functionalized with AS extract); cotton + CH pre-treatment + AS extract (chitosan-pretreated cotton functionalized with AS extract); cotton + CAT pre-treatment + AS extract (cotton pre-treated with a commercial cationic agent and functionalized with AS extract); cotton + AL pre-treatment + AS extract (cotton pre-treated with alum and functionalized with AS extract).

As a first screening, keratinocyte viability was assessed based on metabolic activity following exposure to different AS-functionalized cotton materials after 8 and 24 h under indirect and semi-direct contact conditions (Figure 4A,B). In general, all AS-functionalized textiles—regardless of the extract incorporation method (exhaustion or padding) or the surface pre-treatment applied—maintained keratinocyte viability above the 70% threshold defined by ISO-10993-5 [59] at both time points and under both contact conditions (Figure 4C,D).

Notably, no marked reduction in cell viability was observed following prolonged exposure (24 h) compared to short-term exposure (8 h), indicating the absence of time-dependent cytotoxic effects. While minor variations between exhaustion- and padding-treated textiles with the different pre-treatments (CH, CAT, AL) were detected, these differences did not compromise cell viability and remained within the cytocompatibility range. Collectively, these findings demonstrate the potential biocompatibility of AS-functionalized cotton materials for short and prolonged skin contact applications.

**Figure 5:**
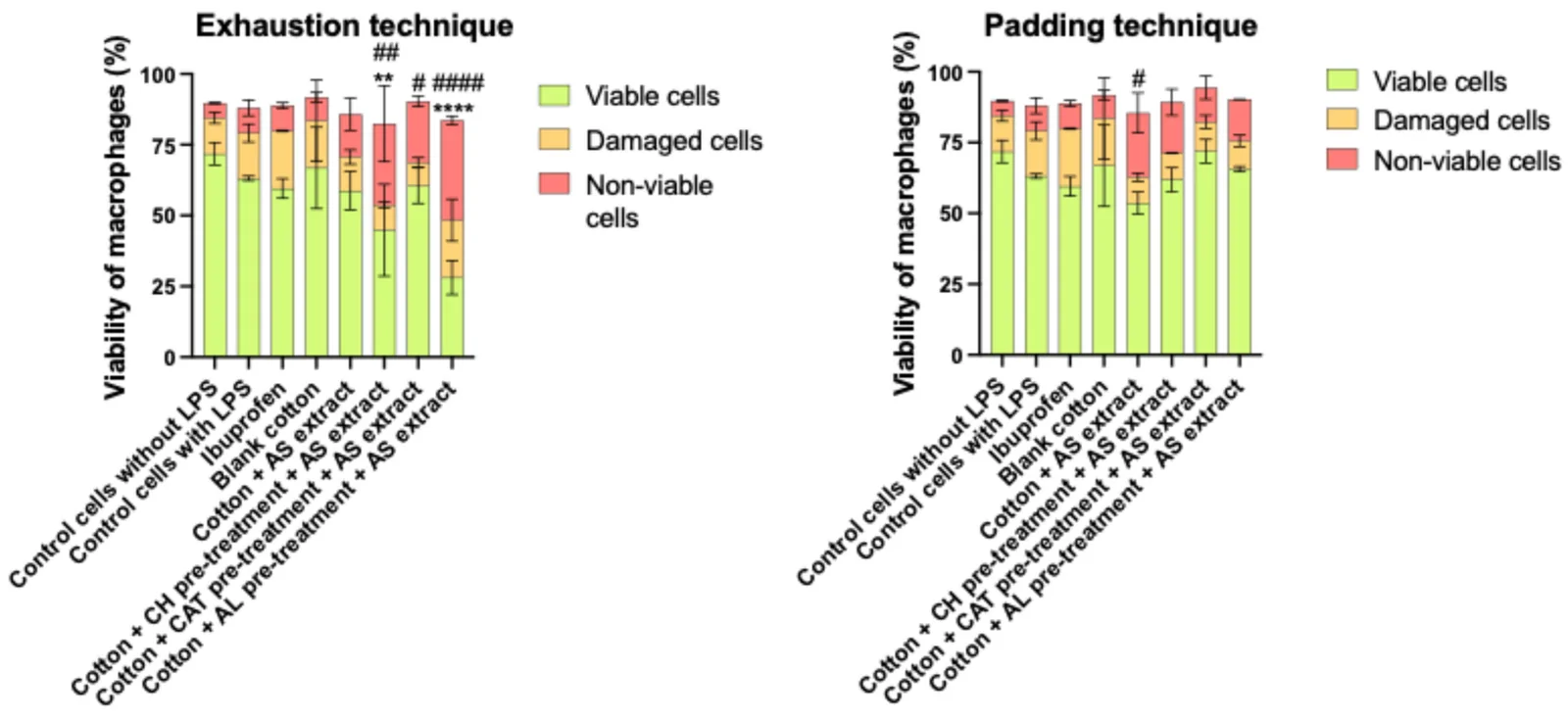
Macrophage viability after 24 h of semi-direct exposure to AS-functionalized cotton materials, assessed by flow cytometry using TO/PI staining. Stacked bar graphs represent the relative distribution (%) of viable (TO^+^/PI^−^), damaged (TO^+^/PI^+^), and non-viable (TO^−^/PI^+^) macrophage populations for each condition. Data are expressed as mean ± SD from three independent experiments. * Indicates statistically significant differences compared to the control without LPS for viable cells, while # denotes statistically significant differences compared to the control without LPS for non-viable cells, where ^#^ = *p* < 0.05, **^, ##^ = *p* < 0.01, ***^, ####^ = *p* < 0.0001.

In contrast to keratinocytes, macrophages exhibited a more heterogeneous response to AS-functionalized cotton materials, as determined by TO/PI flow cytometry analysis (Figure 5). Following 24 h of semi-direct exposure, the viable cells (TO^+^/PI^−^) remained the predominant population under most conditions, accounting for approximately 50–70% of total cells. However, a marked reduction in viability was observed for AS-functionalized cotton pre-treated with CH and AL using the exhaustion method. When compared with the non-LPS-stimulated control, statistically significant differences (*) in the proportion of viable macrophages were detected exclusively for the CH- and AL-pre-treated AS textiles obtained by the exhaustion method, corroborating their comparatively higher impact on macrophage viability.

Concomitantly, a modest increase in compromised macrophage populations was observed relative to the control without LPS (Figure 5). The proportion of damaged cells (TO^+^/PI^+^) remained relatively low, ranging from approximately 5–15% across all conditions. In contrast, the proportion of non-viable cells (TO^−^/PI^+^) showed greater variability among samples. Notably, AS-functionalized cotton prepared via the exhaustion technique—subjected to CH, CAT, or AL pre-treatments—and via the padding technique—cotton functionalized with AS extract but without pre-treatment—induced statistically significant increases (#) in the proportion of non-viable macrophages compared to the control without LPS.

The comparatively higher sensitivity observed in macrophages is biologically plausible and may be attributed to their prolonged culture period (approximately five days from seeding to assay completion) and their intrinsic phagocytic and immune-responsive phenotype, which increases susceptibility to material-derived cues and soluble bioactive compounds from plant extracts. Although elevated PI-positive populations were detected in some conditions—particularly those produced via the exhaustion method—overall macrophage viability remained within acceptable biocompatibility limits. These findings suggest that the observed effects are indicative of limited membrane perturbation rather than severe membrane damage or acute cytotoxicity.

In fact, some studies have demonstrated that textiles enriched with natural extracts, such as aloe vera, turmeric, green tea, onion skin, eucalyptus, and hop, can enhance skin-related functions, including antimicrobial activity, antioxidant protection, and wound healing support, while maintaining acceptable cytocompatibility profiles [37,39,55]. However, multicellular screening involving both keratinocytes and macrophages remains limited.

Some studies have demonstrated that textiles enriched with polyphenol-rich extracts preserve keratinocyte viability when evaluated using metabolic assays such as MTT, PrestoBlue, Calcein-AM/PI, and TO/PI staining [27,39,55]. In this line, our findings show that all AS-functionalized textiles preserved keratinocyte viability above the ISO-defined cut-off at both 8 and 24 h under indirect and semi-direct contact conditions, reinforcing the emerging consensus that textiles incorporating plant extracts can achieve high cytocompatibility in epidermal models.

In contrast to keratinocytes, macrophages often display more variable responses to bioactive materials, including those containing plant extracts, due to their plasticity and sensitivity to material-derived cues [60,61]. Although studies indicate that plant extracts can differentially modulate macrophage membrane integrity and functional activity, largely depending on extract composition and release kinetics [62,63], the use of macrophage models, including primary human macrophages, RAW 264.7 or THP-1–derived cell lines, is still rarely employed to assess the biocompatibility of bioactive textile materials. Consequently, data on the effects of bioactive plant extract-functionalized textiles on macrophages, particularly under inflammatory microenvironmental conditions, remain scarce.

Within this context, the present study demonstrates that macrophage viability remained within acceptable biocompatibility limits, although statistically significant shifts in viable and non-viable subpopulations were observed for selected AS-functionalized textiles, particularly those produced via the exhaustion method with CH, CAT, or AL pre-treatments. Importantly, the proportion of membrane-compromised cells (TO^+^/PI^+^) remained consistently low, indicating limited membrane perturbation rather than extensive cytotoxicity. These alterations are biologically plausible given the phagocytic and immune-responsive nature of macrophages, combined with prolonged in vitro culture, which is known to increase sensitivity to soluble bioactive compounds released from plant extract–functionalized materials.

The distinct responses observed between keratinocytes and macrophages underscore the methodological relevance and novelty of incorporating immune-cell models under inflammatory conditions in the biological evaluation of bioactive textiles. While keratinocyte-based metabolic assays provide a general screen for cytotoxicity, macrophage viability and membrane integrity analyses offer complementary insights into immune compatibility and potential immunomodulatory effects that are not captured by epithelial cells alone.

The multi-cellular and multi-endpoint strategy applied in the present study aligns with best practices in biomaterials research and represents a step beyond conventional textile biocompatibility evaluation. By integrating epithelial and immune cell models, this approach provides a more comprehensive safety profile for materials intended for direct skin contact. Overall, the consistency between our findings and the literature supports the conclusion that AS-functionalized cotton does not induce cytotoxic effects in key skin-relevant cell models.

However, the behavior observed in macrophages is more indicative of complex material–cell interactions than acute toxicity and justify deeper investigation using additional endpoints, including cytokine profiling, oxidative stress analyses, and three-dimensional (3D) skin equivalents in the future. Such studies will be crucial to fully define the biocompatibility and immunological footprint of plant extract–derived bioactive textiles in translational contexts.

### 3.4. Anti-Inflammatory Activity of AS-Functionalized Cotton

The evaluation of anti-inflammatory properties in bioactive textiles is increasingly recognized as essential, as skin-contact materials can be designed to modulate local immune responses and benefit dermatological conditions driven by inflammation [64,65]. When combined with antimicrobial, antioxidant, and biocompatible properties, these activities can significantly enhance patient comfort and clinical performance, particularly in applications targeting skin microbiota dysregulation.

**Figure 6:**
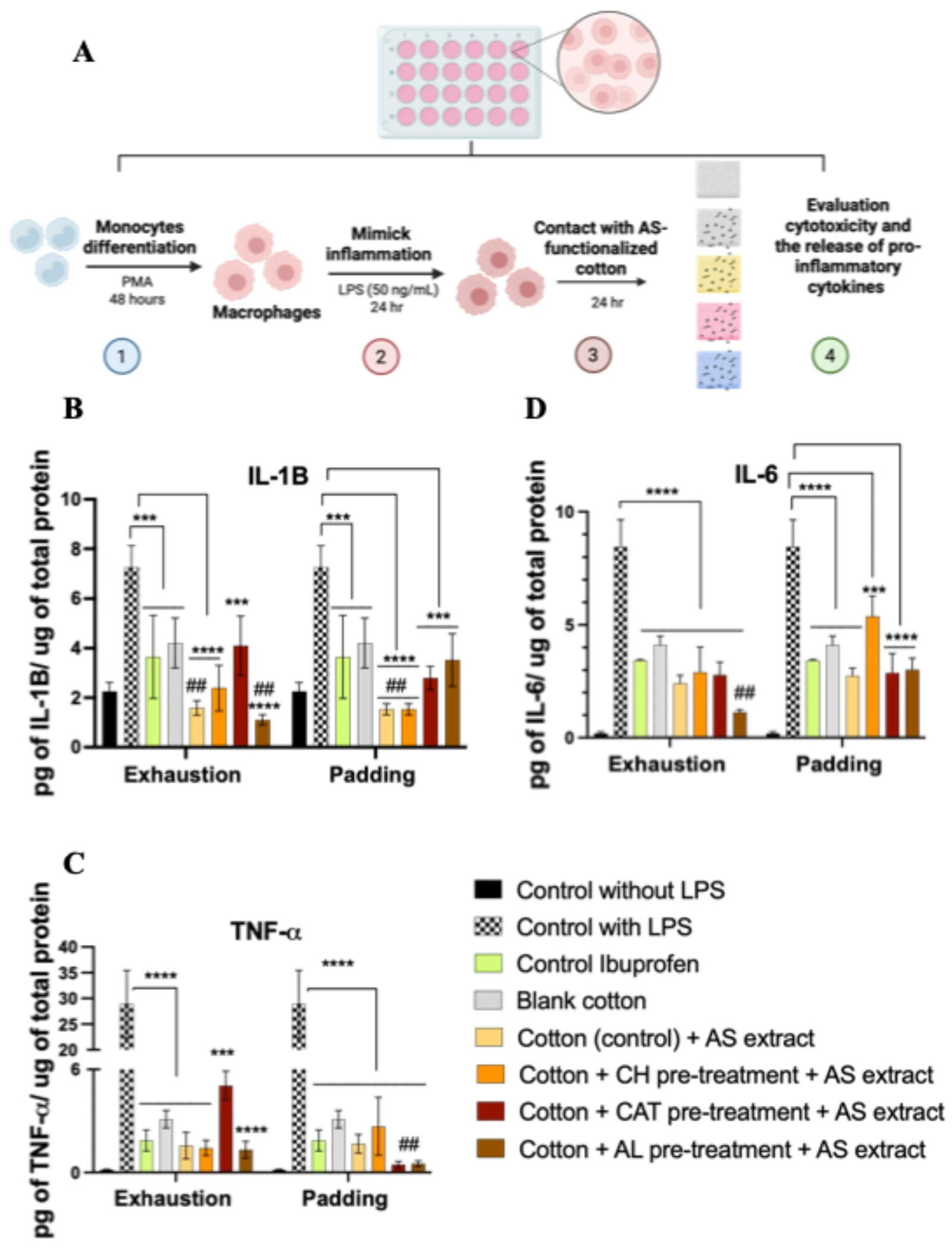
(**A**) Schematic representation of the experimental steps used to assess the anti-inflammatory response of macrophages exposed to the AS-functionalized cotton materials. (1) Differentiation of monocytes into macrophages. (2) Stimulation of macrophages with LPS to induce an in vitro pro-inflammatory environment. (3) Exposure of macrophages to ibuprofen, non-functionalized and AS-functionalized cotton materials for 24 h, and (4) Evaluation of cytotoxicity and the release of pro-inflammatory cytokines. (**B**–**D**) Anti-inflammatory activity of AS-functionalized cotton in LPS-stimulated macrophages. Levels of IL-1β, TNF-α, and IL-6 were quantified in culture supernatants of monocyte-derived macrophages following 24 h exposure to AS-functionalized cotton samples with previous LPS stimulation. Two functionalization strategies were used for incorporation of AS extract: exhaustion and padding. Within each method, cotton was tested without pre-treatment (Cotton Control), or after pre-treatment with CH, AL or CH, followed by incorporation of AS extract. Unstimulated cells (Control without LPS) and LPS-stimulated cells (Control with LPS) served as negative and positive inflammation controls, respectively, while ibuprofen was included as a pharmacological anti-inflammatory reference. Data are presented as mean ± SD (n = 3). * Indicates significant differences compared with the LPS control; # indicates significant differences compared with ibuprofen, where ^##^ = *p* < 0.01, *** = *p* < 0.001, **** = *p* < 0.0001.

To investigate the anti-inflammatory potential of AS-functionalized cotton, we employed a macrophage model differentiated from human monocytes (THP-1) and stimulated with LPS to mimic an inflamed skin microenvironment (Figure 6A). This approach enables the assessment of textile-mediated immunomodulation under conditions that closely resemble cutaneous inflammatory responses.

The pro-inflammatory cytokines evaluated (IL-6, IL-1β, TNF-α) are involved in the initiation and amplification of inflammatory responses. IL-1β and TNF-α are rapidly produced by activated monocytes/macrophages and play central roles in triggering inflammation, promoting endothelial activation, leukocyte recruitment, and the expression of additional inflammatory mediators [66]. IL-6 acts both as a pro- and anti-inflammatory cytokine and is critically involved in the acute-phase response, immune cell differentiation, and the transition from acute to chronic inflammation [67]. The quantification of these cytokines provides valuable insight into the inflammatory status of cells exposed to AS-functionalized cotton.

In fact, the stimulation with LPS markedly increased the levels of all pro-inflammatory cytokines evaluated—IL-1β, TNF- α, and IL-6—when compared to unstimulated macrophages (control without LPS), as demonstrated in Figure 6. These results confirm the successful induction of an acute pro-inflammatory response in vitro. In contrast, treatment with ibuprofen significantly reduced these LPS-induced increases and was therefore used as a reference of anti-inflammatory activity.

In general, exposure to AS-functionalized cotton samples significantly reduced the release of all pro-inflammatory cytokines analyzed—IL-1β, TNF, and IL-6—when compared with the LPS-stimulated control (*). Interestingly, in several experimental conditions, the magnitude of this reduction was comparable to, or surpassed the anti-inflammatory effect of ibuprofen (#), used as a positive anti-inflammatory control.

More specifically, for IL-1β, all AS-functionalized cotton samples, irrespective of the surface pre-treatment strategy or the functionalization method used for AS incorporation, significantly decreased cytokine levels relative to the LPS group (*p* ≤ 0.001 to ≤0.0001; Figure 6B). Notably, cotton control with AS extract and cotton pre-treated with AL followed by AS incorporation via exhaustion demonstrated a significantly greater inhibitory effect than ibuprofen (*p* ≤ 0.01). A similar enhancement was observed for cotton control with AS extract and cotton pre-treated with CH when AS was incorporated using the padding technique. These findings indicate that certain AS-functionalized textiles can attenuate IL-1β release beyond the pharmacological reference.

For TNF, AS-functionalized cotton induced a consistent and significant reduction in cytokine release compared to the LPS control (*p* ≤ 0.001 to ≤0.0001; Figure 6C). Although ibuprofen effectively decreased TNF levels, several AS-based conditions achieved comparable or slightly greater reductions. These findings indicate that TNF modulation by these bioactive textiles is robust across different functionalization approaches, particularly in AS-functionalized samples produced using the padding method and pre-treated with CAT and AL (*p* ≤ 0.01).

Similarly, for IL-6, exposure to AS-functionalized samples led to a marked decrease in cytokine secretion relative to the LPS-stimulated macrophage control (*p* ≤ 0.001 to ≤0.0001; Figure 6D). In several conditions, the inhibitory effect matched or exceeded that of ibuprofen, with a more pronounced reduction observed in samples obtained via the exhaustion method and pre-treated with Al (*p* ≤ 0.01). Consistent with the previous results, AS incorporation through the exhaustion method yielded the most effective outcomes, reinforcing the influence of the functionalization strategy in determining the bioactivity of these materials, including their anti-inflammatory potential.

Together, these results demonstrate that AS-functionalized cotton textiles effectively mitigate LPS-induced inflammatory signaling across multiple cytokines. The fact that several samples outperformed ibuprofen highlights their promising therapeutic potential and supports the development of bioactive textiles with incorporation of AS extract as modulators of cutaneous inflammation.

At the molecular level, the observed reduction in IL-1β, TNF-α, and IL-6 suggests that AS-functionalized cotton textiles may interfere with key intracellular signaling pathways triggered by LPS stimulation. LPS is known to activate macrophages primarily through Toll-like receptor 4 (TLR4), leading to downstream activation of the NF-κB and MAPK pathways, which are central regulators of pro-inflammatory gene expression [68]. The marked attenuation of early cytokines such as IL-1β and TNF-α indicates a possible inhibition of NF-κB nuclear translocation and/or suppression of upstream kinases involved in MyD88-dependent signaling. In parallel, the significant reduction in IL-6 secretion suggests modulation of downstream inflammatory cascades, potentially involving MAPK (p38, ERK, JNK) and STAT3 signaling pathways [33,69]. Although these mechanisms were not directly investigated in the present study, the cytokine profile observed strongly supports the involvement of these inflammatory pathways.

These results can be attributed in part to the phenolic compounds present in AS extracts, which are well known for their anti-inflammatory activity, largely mediated by the downregulation of pro-inflammatory cytokines. This effect is mainly attributed to the inhibition of TLR4-dependent NF-κB and MAPK signaling pathways, resulting on the suppression of inflammatory gene expression [69,70].

Although recent reviews and experimental studies have reported rapid advances in the antimicrobial and antioxidant properties of bioactive textiles, research addressing their anti-inflammatory potential remains comparatively limited. In particular, studies focusing on broad cytokine screening, underlying mechanisms of action, standardized evaluation methods, and in vivo validation are still scarce.

For instance, ibuprofen-loaded coatings, ibuprofen-functionalized silica nanoparticles, and several nanostructured systems (e.g., zinc oxide, titanium oxide and silver oxide nanoparticles, as well as silver nanocolloids) have been applied to develop fabrics capable of reducing pro-inflammatory markers such as IL-1β and TNF-α in vitro [71,72,73]. However, these studies conducted using fibroblasts or leukocytes alone, may not accurately reflect the real anti-inflammatory performance of these materials, given the complexity of human skin. They often overlook the role of keratinocytes—the primary cells in direct contact with such materials—as well as macrophages, which are key regulators of the skin immune response.

Moreover, such reports frequently prioritize material formulation, biophysical properties, drug release, or antimicrobial performance rather than employing standardized inflammatory readouts, such as broader cytokine panels, LPS-stimulated models, or clinically relevant endpoints. As a result, comparisons with well-established textile materials with anti-inflammatory properties are still largely missing.

This gap is particularly evident for bioactive textiles incorporating plant-derived extracts, where only a limited number of studies have investigated their anti-inflammatory potential [55,74]. This lack of investigation contrasts with extensive evidence demonstrating the in vitro anti-inflammatory activity of plant extracts, including AS extracts, either used alone or incorporated into formulated systems [20,69,70,75].

In this context, the present study contributes to addressing this unmet need by providing a broader evaluation of cytokine release using LPS-stimulated macrophages, an in vitro model representative of the inflammatory microenvironment. By demonstrating the simultaneous downregulation of key pro-inflammatory mediators—each playing distinct and complementary roles in the initiation and propagation of inflammation—these findings highlight the potential of bioactive textiles incorporating plant extracts rich in polyphenolic compounds to modulate multiple stages of the inflammatory response at the skin-textile interface.

This effect is particularly relevant for topical dermatological applications, where direct interaction between the textile and the skin may enable the local release of bioactive compounds capable to modulate local inflammatory responses. Considering that skin microbiota dysregulation is frequently associated with persistent local inflammation, the ability of AS-functionalized cotton to reduce the release of pro-inflammatory cytokines may contribute to restore a more balanced skin microenvironment and alleviating inflammation-associated skin dysfunction. Therefore, the observed anti-inflammatory activity should be interpreted as a local effect resulting from the topical application of the textile and its interaction with the immune cells of skin, rather than a systemic anti-inflammatory action. These findings further support the potential use of AS-functionalized cotton as a bioactive skin-contact textiles for the management of skin conditions associated with microbiota imbalance and inflammation.

### 3.5. Phenolic Compounds Release from AS-Functionalized Cotton Under Skin-Mimicking Conditions

Despite the growing interest in bioactive textiles functionalized with plant-derived extracts rich in phenolic compounds, the quantitative evaluation of phenolic loading and release behavior remains largely underexplored. This knowledge gap is particularly relevant for skin-contact applications, where the local concentration, bioavailability, and release kinetics of active compounds are key determinants of both therapeutic efficacy and safety.

**Figure 7:**
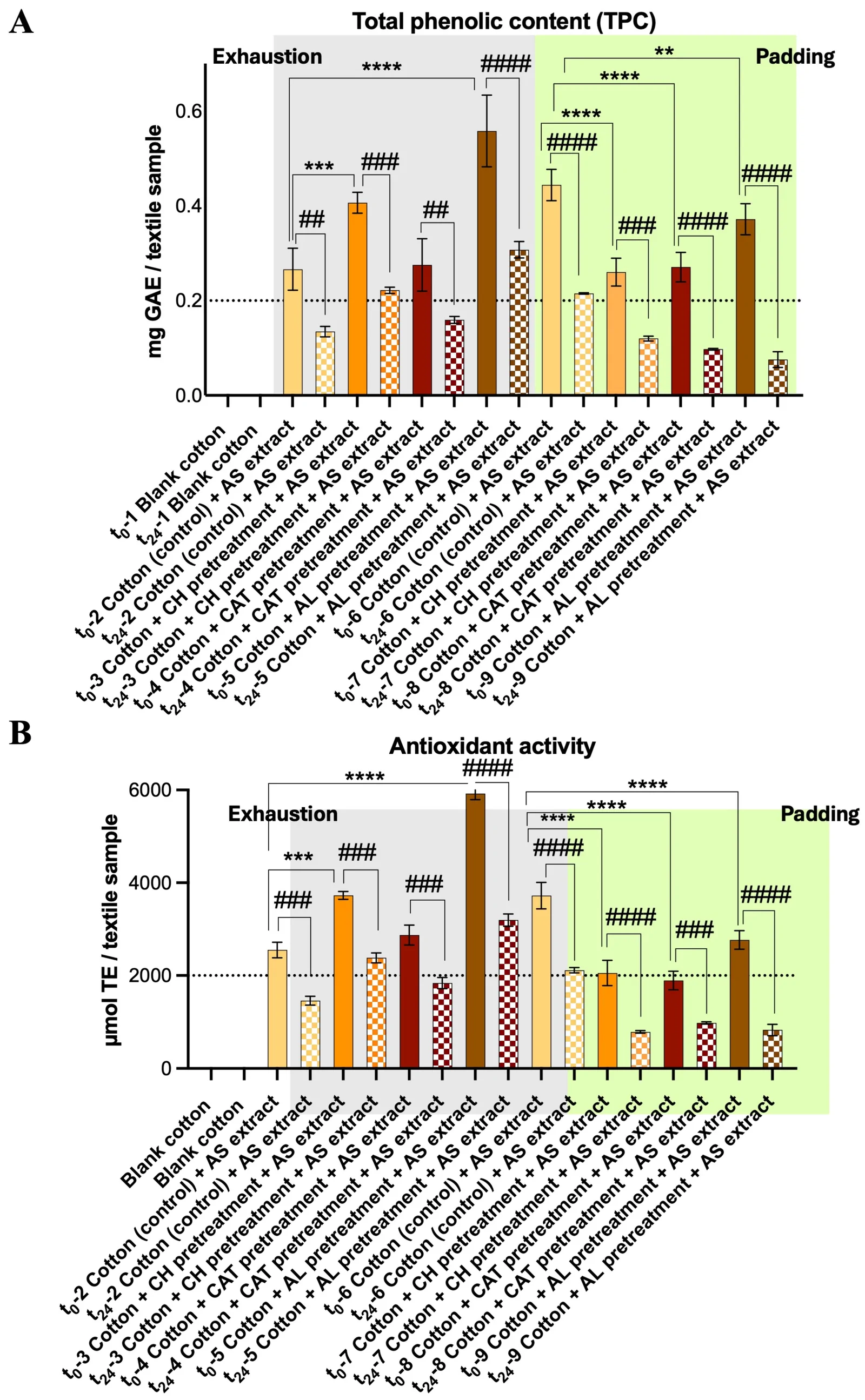
(**A**) Quantification of total phenolic compounds (TPC) and (**B**) evaluation of antioxidant activity of AS-functionalized cotton materials. For both, samples were analyzed at baseline (t_0_) and after 24 h of exposure to artificial human sweat (t_24_). Grey panels correspond to cotton functionalized by the exhaustion method, while green panels represent cotton functionalized by padding. Bars represent mean ± SEM for each treatment group; solid colors and corresponding patterned bars indicate the same condition of cotton evaluated at t_0_ and t_24_, respectively. Statistical significance is indicated as follows: * indicates significant differences compared with cotton (control) subjected to the same AS extract functionalization method; # indicates significant differences between the same sample at t_0_ and t_24_. Significance levels are expressed *p* < 0.01, *p* < 0.001, and *p* < 0.0001, respectively, for **/^##^; ***/^###^; ****/^####^.

In this context, the release of phenolic compounds from AS-functionalized cotton textiles was investigated under simulated physiological conditions to elucidate the contribution of AS-derived bioactive compounds to the overall material performance when in direct contact with artificial human sweat—the same conditions used in the indirect method for biocompatibility evaluation. The TPC initially incorporated into the textiles (t_0_), as well as the fraction released into artificial human sweat after 24 h (t_24_), were quantified using the Folin–Ciocalteu assay (Figure 7A). In parallel, the antioxidant activity of these materials was assessed to determine whether the released phenolic compounds retained their functional bioactivity upon diffusion into the surrounding medium (Figure 7B).

As shown in Figure 7A, all AS-functionalized cotton samples exhibited measurable phenolic content at t_0_ and a consistent decrease at t_24_, confirming the release of phenolic compounds into the artificial sweat solution. Blank cotton samples displayed negligible TPC values at both time points, validating that the detected phenolics originated exclusively from the incorporated AS extract. The initial phenolic loading ranged from approximately 0.260 to 0.558 mg GAE per textile sample, with exhaustion-treated samples (2–5) showing higher TPC values than padding-treated textiles (6–9), indicating more efficient incorporation. Among all conditions, the AL pre-treated sample obtained by exhaustion (sample 5) exhibited the highest loading, highlighting the beneficial effect of this pre-treatment in enhancing fibre-polyphenol interactions.

Notably, pre-treatment strategies had distinct effects depending on the functionalization method. When combined with the exhaustion method, all cationized samples (3–5) showed higher phenolic incorporation compared to the control (sample 2) with CH and AL pre-treatments showing statistically significant increases (*p* < 0.001 and *p* < 0.0001). However, the opposite trend was observed for padding-treated samples (7–9), which exhibited lower TPC relative to their control (sample 6), with all the pre-treated samples differing significantly (*p* < 0.001 and *p* < 0.0001). These results reinforce the importance of the interaction between processing method and surface chemistry in determining phenolic uptake.

Comparison between t_0_ and t_24_ indicates that phenolic release generally increases with initial loading; however, it is also governed by the strength of interactions between phenolic compounds and the textile matrix. Exhaustion-treated samples exhibited higher initial TPC and correspondingly greater release after 24 h (≈50–58%), with statistical differences highlighted in Figure 7A. This behavior suggests that, despite higher uptake, phenolic compounds are more readily released upon contact with human sweat, indicating more reversible or weaker binding interactions in exhaustion-treated systems, particularly for AL pre-treated samples (*p* < 0.0001). In contrast, padding-treated samples showed significantly lower release levels (≈20–40%), with all pre-treated samples differing statistically from their respective control (*p* < 0.001–0.0001). This trend is consistent with a more superficial deposition but comparatively stronger retention or less desorption under the same conditions.

Overall, these results demonstrate that although initial loading influences the extent of release, the dominant factor controlling phenolic release is the interaction strength between the compounds and the textile substrate, as supported by the significant differences observed between treatment methods.

This balance between retention and release is critical, as it may directly influence the bioavailability of phenolic compounds at the skin interface. In this regard, AS-functionalized cotton can be understood as a reservoir system, where processing parameters dictate whether the material favors a rapid or sustained delivery of bioactive compounds.

Importantly, when correlating TPC with antioxidant activity at both t_0_ and t_24_, a strong and statistically significant relationship was observed, confirming that phenolic compounds retained their radical-scavenging capacity after both incorporation and release (Figure 7B).

Blank cotton samples exhibited negligible antioxidant activity, further supporting that the measured activity arises exclusively from the incorporated AS-derived phenolic compounds.

Exhaustion-treated samples showed higher antioxidant activity at t_0_, reflecting their greater phenolic loading, whereas padding-treated samples exhibited lower initial activity. Comparison between t_0_ and t_24_ revealed a statistically significant decrease in antioxidant activity for both exhaustion- and padding-treated samples (*p* < 0.001 and *p* < 0.0001). Notably, this reduction correlates with their release profiles and the corresponding decrease in phenolic compounds remaining in the AS-functionalized cotton samples. Despite this decline, all functionalized samples retained measurable antioxidant activity. These results indicate that the released phenolic compounds remain functionally active in solution and that their antioxidant performance is closely linked to their availability, as supported by the statistically significant trends observed across treatments and time points.

Beyond the antioxidant activity assessed under the same conditions for TPC quantification, the release behavior of phenolic compounds provides a mechanistic basis for the multifunctional biological activities observed throughout this study. The antimicrobial effects, particularly against Gram-positive bacteria, can be attributed to the availability of phenolic compounds capable of disrupting bacterial membranes, complexing proteins, and interfering with microbial metabolism. In general, this activity is closely linked to the amount of phenolic compounds incorporated and their subsequent release profiles, which differ according to the functionalization method applied. Exhaustion-treated samples, which exhibit greater phenolic uptake and enhanced release, are expected to exhibit a strong antimicrobial effect. In contrast, padding-treated samples, with lower phenolic loading and more restricted release, are likely to show a reduced antimicrobial activity. A consistent trend is also observed in the antioxidant activity, where DPPH results indicate enhanced performance in samples functionalized via the exhaustion method. This reinforces the relationship between higher phenolic incorporation, release rate, and improved bioactivity. Overall, these findings demonstrate that both antimicrobial and antioxidant performances are primarily governed by the efficiency of phenolic incorporation and the dynamics of their subsequent release from the treated cotton matrices.

Similarly, the anti-inflammatory activity observed in LPS-stimulated macrophages is consistent with the release of bioactive phenolics into the surrounding medium. These compounds are known to modulate key inflammatory pathways, including NF-κB and MAPK signaling, resulting in reduced secretion of pro-inflammatory cytokines such as IL-1β, TNF-α, and IL-6. The significant reduction in these cytokines—often comparable to or exceeding that of ibuprofen—indicates that the released phenolic fraction is biologically available and active at the cellular level. In this context, controlled release appears particularly advantageous, as it may enable sustained immunomodulatory effects without excessive exposure.

From a safety perspective, the biocompatibility results further support the relevance of controlled phenolic release. All AS-functionalized textiles maintained keratinocyte viability above the ISO-defined threshold, indicating that the concentration of released compounds remains within safe limits for skin cells. However, the slightly higher sensitivity observed in macrophages, particularly for exhaustion-treated samples with higher phenolic loading (e.g., CH and AL pre-treated sample), highlights the importance of tuning release profiles to balance bioactivity and cellular compatibility. Importantly, these effects did not indicate acute cytotoxicity but rather suggest biologically relevant interactions between released compounds and immune cells.

Collectively, these results show that AS-functionalized cotton textiles may act as effective reservoirs of phenolic compounds, capable of delivering bioactive molecules in a controlled manner under skin-mimicking conditions. The release profiles are strongly governed by the interplay between functionalization method and pre-treatment strategy, which dictate the balance between phenolic retention within the fibre matrix and their subsequent availability at the interface. This delivery rate is directly reflected in the correlated antioxidant, antimicrobial, and anti-inflammatory responses, highlighting that not only the TPC but also its release kinetics are critical determinants of biological performance.

Furthermore, the findings validate the relevance of TPC quantification as a predictive tool of functional performance—an aspect still underexplored in bioactive textile research—and demonstrate that processing parameters play a pivotal role in modulating both phenolic incorporation and release behavior. More importantly, this study provides clear evidence that tailoring these parameters enables the design of textile-based delivery systems with tunable bioactivity profiles, opening new opportunities for the development of safe and effective skin-contact materials with sustained multifunctional properties.

Additionally, a key aspect for the practical implementation of these materials is the durability of their functional properties after washing. In this line, all AS-functionalized cotton textile demonstrated that, in general, their biological activities decrease significantly after short domestic washing cycles, reflecting the strong release of phenolic compounds from the textile substrates. These findings are consistent with the release behavior reported in this section, where a substantial fraction of the incorporated phenolic compounds was released after 24 h of contact with artificial sweat. Together, these findings highlight the need to improve functionalization strategies capable of enhancing the retention of AS-derived bioactive compounds within cotton fibres to preserve their biological efficacy after domestic washing cycles.

## 4. Conclusions

From a broader perspective, the novelty of this work lies in the integration of multiple biological endpoint validation with quantitative phenolic release profiling under relevant physiological skin conditions for the evaluation of AS-functionalized cotton materials. While most studies in this field focus either on extract characterization or isolated functional properties, this work provides a direct correlation between biological performance, chemical composition, and release behavior. Furthermore, the use of artificial human sweat as a release medium, combined with skin-relevant cellular models, offers a more realistic approximation of in-use conditions, which remains scarce in the literature.

Overall, AS-functionalized cotton textiles emerge as tunable bioactive systems, where industrially relevant processes such as exhaustion and padding, combined with targeted pre-treatment strategies, can be strategically employed to design materials with controlled and predictable biological responses for local skin applications. This approach represents a significant advancement in the rational development of sustainable, multifunctional, and skin-compatible bioactive textiles for dermatological applications. These materials show strong potential as antimicrobial, antioxidant, and anti-inflammatory platforms for managing skin microbiota dysregulation. However, to effectively translate AS-functionalized cotton for the management of skin dysbiosis conditions, further studies are required to stablish a systematic dose–response relationship, evaluate their long-term stability and durability after washing cycles, and explore their biological performance in physiologically relevant three-dimensional (3D) skin models.

## Data Availability

The data supporting the findings of this study are available within the article and Supplementary Material. However, all data related to this work are available from the corresponding author upon reasonable request.

## Supplementary Materials

The following supporting information can be downloaded at: https://www.mdpi.com/article/10.3390/pharmaceutics18101301/s1, Figure S1: Scanning Electron Microscopy (SEM) images of untreated cotton (blank) and cotton fabrics functionalized with acorn shell (AS) extract using exhaustion and padding techniques. The images show the surface morphology of cotton fibers without functionalization and after AS incorporation, both in the absence and presence of the different pre-treatment conditions. Representative images were acquired at a magnification of 2500×, allowing visualization of morphological changes associated with the functionalization process and extract deposition on the fiber surface. Blank cotton (untreated and non-functionalized cotton); cotton (control) + AS extract (untreated cotton functionalized with AS extract); cotton + CH pre-treatment + AS extract (chitosan pretreated cotton functionalized with AS extract); cotton + CAT pre-treatment + AS extract (cotton pre-treated with a commercial cationic agent and functionalized with AS extract); cotton + AL pre-treatment + AS extract (cotton pre-treated with alum and functionalized with AS extract).

## Abbreviations

ABTS	2,2′-azinobis(3-ethylbenzothiazoline-6-sulphonic acid
AD	Atopic dermatitis
AL	Potassium alum
AS	Acorn shell
CAT	Commercial cationic agent
CFU	Colony-forming units
CH	Chitosan
DMEM	Dulbecco’s Modified Eagle Medium
DPPH	2,2-diphenyl-1-picrylhydrazyl
FBS	Fetal Bovine Serum
GAE	Gallic acid
HaCaT	Human keratinocyte cell line
LPS	Lipopolysaccharide
MTT	3-(4,5-dimethylthiazol-2-yl)-2,5-diphenyltetrazolium bromide
PI	Propidium Iodide
PMA	Phorbol-12-myristate-13-acetate
RT	Room temperature
THP-1	Human monocyte leukemia cell line
TO	Thiazole Orange
TPC	Total Phenolic Content
TSA	Tryptic Soy Agar
TSB	Tryptic Soy Broth
