# Supplementary material for: Acorn Shell Extract-Functionalized Cotton Biomaterials: A Sustainable Platform with Biological Activities and Controlled Release for Skin Microbiota Dysregulation Conditions
Source: Pharmaceutics. 2026 Oct 14;18(10):1301. doi: 10.3390/pharmaceutics18101301 (PMC13708666; doi:10.3390/pharmaceutics18101301)
Supplement: Supplementary file 1 [file pharmaceutics-18-01301-s001.zip › pharmaceutics-4553828-supplementary.pdf]

Article

Acorn Shell Extract-Functionalized Cotton Biomaterials: A Sustainable Platform with Biological Activities and Controlled Release for Skin Microbiota Dysregulation Conditions

Cláudia S Oliveira [1,2,*], Andreia Fernandes [1,3], Adma Melo [1], Tiago B Afonso [1], Ana F Costa [4], Manuela Pintado [1], Carla Silva [4], and Freni K Tavaria [1]

[1] Universidade Católica Portuguesa, CBQF - Centro de Biotecnologia e Química Fina – Laboratório Associado, Escola Superior de Biotecnologia, Rua Diogo Botelho 1327, 4169-005 Porto, Portugal; admelo@ucp.pt; tbafonso@ucp.pt; mpintado@ucp.pt; ftavaria@ucp.pt

[2] RISE-Health - Faculty of Medicine, University of Porto, Alameda Prof. Hernâni Monteiro, 4200-319, Porto, Portugal; csfoliveira@med.up.pt

[3] LAQV – REQUIMTE, Departamento de Química e Bioquímica, Faculdade de Ciências, Universidade do Porto, Rua do Campo Alegre 687, 4169-007, Porto, Portugal; s-ansifernandes@ucp.pt

[4] Technological Centre of the Textile and Clothing Industries, CITEVE – Rua Fernando Mesquita 2785, 4760-034 Vila Nova de Famalicão, Portugal; afcosta@citeve.pt; cjsilva@citeve.pt

* Correspondence: csfoliveira@med.up.pt

Supplementary Material

To evaluate the surface morphology of the AS-functionalized cotton, the samples were mounted on observation pins using double-sided adhesive carbon tape (NEM tape, Nisshin, Japan) and sputter-coated with a 9–12 nm layer of gold/palladium (Polaron, Germany) to improve surface conductivity. Afterwards, morphological analyses were examined using a Phenom Pro G6 scanning electron microscope (SEM) (Thermo Fisher Scientific, Eindhoven, The Netherlands) operated at an accelerating voltage of 10 kV under the low-vacuum conditions (1.0 pa). For better visualization of the surface topography, images were acquired using the secondary electron detector (SED), at magnification of 2500x.

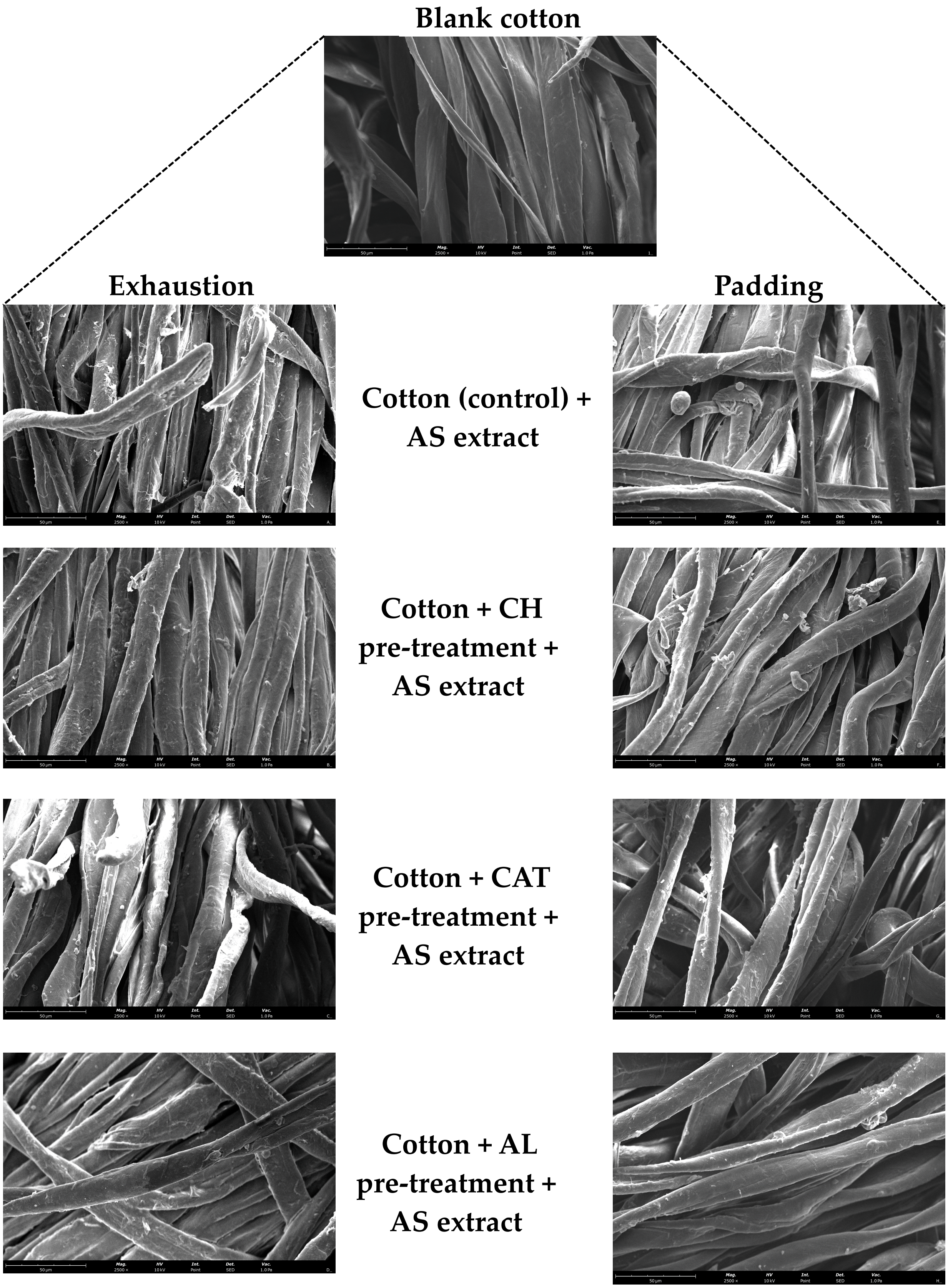

Blank cotton
Exhaustion
Padding
Cotton (control) + AS extract
Cotton + CH pre-treatment + AS extract
Cotton + CAT pre-treatment + AS extract
Cotton + AL pre-treatment + AS extract

Figure S1: Scanning Electron Microscopy (SEM) images of untreated cotton (blank) and cotton fabrics functionalized with acorn shell (AS) extract using exhaustion and padding techniques. The images show the surface morphology of cotton fibers without functionalization and after AS incorporation, both in the absence and presence of the different pre-treatment conditions. Representative images were acquired at a magnification of 2500×, allowing visualization of morphological changes associated with the functionalization process and extract deposition on the fiber surface. Blank cotton (untreated and non-functionalized cotton); cotton (control) + AS extract (untreated cotton functionalized with AS extract); cotton + CH pre-treatment + AS extract (chitosan pretreated cotton functionalized with AS extract); cotton + CAT pre-treatment + AS extract (cotton pre-treated with a commercial cationic agent and functionalized with AS extract); cotton + AL pre-treatment + AS extract (cotton pre-treated with alum and functionalized with AS extract).
